# Genome-Wide Analysis of Ammonium Transporter Genes in Flowering Chinese Cabbage and Functional Insights into BcAMT1.1 Under Low-Nitrogen Conditions

**DOI:** 10.3390/plants14243812

**Published:** 2025-12-14

**Authors:** Yunna Zhu, Lihua Zhong, Qiuxiang Zhong, Xinmin Huang, Ali Anwar, Wei Su, Riyuan Chen, Shiwei Song

**Affiliations:** 1 College of Horticulture, South China Agricultural University, Guangzhou 510642, China; 2 College of Biology and Agriculture, Shaoguan University, Shaoguan 512005, China; 3 College of Agriculture and Food Engineering, Baise University, Baise 533000, China; 4 Guangdong Provincial Key Laboratory for Green Agricultural Production and Intelligent Equipment, College of Biology and Food Engineering, Guangdong University of Petrochemical Technology, Maoming 525000, China

**Keywords:** ammonium transporter, flowering Chinese cabbage, low-nitrogen conditions, genome-wide analysis

## Abstract

As a primary macronutrient, nitrogen is integral to plant growth and regulates their development; ammonium transporters (AMTs) mediate nitrogen absorption and its involvement in metabolism. In this study, nine *BcAMT* genes were identified in flowering Chinese cabbage (*Brassica campestris*) and were systematically categorized into two subfamilies. Their evolutionary relationships, conserved motifs, chromosomal distribution, cis-regulatory elements, and expression profiling were systematically characterized. RNA sequencing and quantitative real-time PCR (qRT-PCR) analyses demonstrated that *BcAMT1.1* was abundantly expressed in roots, leaves, and stems of flowering Chinese cabbage and was markedly upregulated under nitrogen deficiency. Assessing subcellular location using GFP fusion demonstrated that BcAMT1.1 localized to the plasma membrane. Functional assays identified heterologous expression in the yeast mutant strain 31019b, and transgenic *Arabidopsis* validated that BcAMT1.1 acted as a functional ammonium transporter. Compared with the wildtype, overexpressing *BcAMT1.1* promoted seedling growth, enhanced NH_4_^+^ influxes and NO_3_^−^ effluxes under low-nitrogen conditions, and significantly increased the transcription levels of key nitrogen assimilation genes (i.e., *AtGLN1.1*, *AtGLN2*, *AtGDH2*). Collectively, our findings enhance the fundamental understanding of *BcAMT* gene functions and highlight BcAMT1.1 as a crucial component in nitrogen uptake and assimilation under low-nitrogen conditions, providing valuable genetic resources for improving nitrogen efficiency in vegetable crops.

## 1. Introduction

Nitrogen, a critical macronutrient, is indispensable for plant physiological functioning and responsible for up to 50% of biomass accumulation [1]. Due to its significant added value, nitrogen fertilizers are often applied in excess to meet actual crop demands, providing low-cost insurance against potential yield losses [2,3]. In vegetable crop production, over-application of nitrogen, combined with low recovery and intensive irrigation, markedly reduces nitrogen use efficiency (NUE) and generates dual environmental and health risks, including water pollution, greenhouse gas emissions, and excessive accumulation of nitrates in edible plant organs, particularly in leafy vegetables [2]. Thus, improving NUE is essential for sustainable agricultural production and relies on a thorough understanding of the mechanisms governing nitrogen absorption, transport, and metabolism.

Ammonium (NH_4_^+^) and nitrate (NO_3_^−^) are the main inorganic nitrogen sources utilized by plants [4]. In soils, NH_4_^+^ concentrations (20 to 200 μmol·L^−1^) are lower than those of NO_3_^−^ (100 μmol·L^−1^ to 70 mmol·L^−1^) [5,6]. Although NO_3_^−^ is generally the predominant nitrogen form for most plants, NH_4_^+^ may be preferred under low-nitrogen conditions or high carbon dioxide levels, because it requires less energy for assimilation via the glutamine synthetase/NADH-dependent glutamate synthase (GS/GOGAT) pathway [7,8]. Nevertheless, excessive NH_4_^+^ can be toxic to plants [6,9]. Therefore, NH_4_^+^ uptake and transportation need to be strictly regulated. NH_4_^+^ uptake in plants is mediated by two distinct systems: the high-affinity transport system (HATS) and the low-affinity transport system (LATS). At low-NH_4_^+^ concentrations (<1 mmol·L^−1^), NH_4_^+^ uptake is primarily mediated by HATS, ensuring efficient NH_4_^+^ absorption, whereas LATS is active at higher NH_4_^+^ concentrations [10,11,12]. Ammonium transporters (AMTs), members of the AMT/methylammonium permease (MEP)/rhesus (Rh) protein family, are key mediators of NH_4_^+^ uptake and transport in plants [11,13]. AMT proteins, mainly found in plants, are grouped into two major subfamilies: the AMT1 subfamily, comprising only the AMT1 cluster, and the AMT2 subfamily, including clusters AMT2, AMT3, and AMT4 [14]. AMT2 has high homology for yeast MEP and *Escherichia coli* AmtB but is less homologous to AMT1 proteins [15].

Following the identification of the first *AMT* in *Arabidopsis* [16], AMT members have subsequently been reported in various species, including rice [17,18], rapeseed [19], soybean [20], and tobacco [21]. In *Arabidopsis*, six AMTs have been isolated and characterized, including five members of the AMT1 subfamily and a single AMT2 isoform [22,23]. Among them, *AtAMT1.1*~*AtAMT1.3* and *AtAMT1.5* are primarily expressed in the roots and are strongly induced by nitrogen deficiency; functional analyses have shown that AtAMT1.1–AtAMT1.3 contribute nearly 90% of plants’ high-affinity NH_4_^+^ uptake capacity, with AtAMT1.1 and AtAMT1.3 accounting for approximately 65~70% of the total; AtAMT1.2 accounting for 18~26% of the total; and AtAMT1.5 accounting for 5~10% of the total [22]. AtAMT1.4 is a pollen-specific AMT member and mediates NH_4_^+^ absorption in pollen [24]. In rice, 12 AMTs are isolated; OsAMT1.1~OsAMT1.3 account for up to 95% of high-affinity NH_4_^+^ uptake [18], and the knockout of *OsAMT1.1* reduces NH_4_^+^ uptake by 25~30% [25]. In rapeseed, twenty *BnaAMT* genes were identified, including fourteen members in the AMT1 subfamily and six members in the AMT2 subfamily, most of which are highly responsive to external nitrogen conditions [19]. Together, these findings indicate that AMT1, particularly AMT1.1 and AtAMT1.3, are involved in high-affinity NH_4_^+^ uptake in plants.

Flowering Chinese cabbage (*Brassica campestris* L. ssp. *chinensis* var. *utilis* Tsen et Lee) is a typical stalk vegetable from South China that has significant health and economic value [26,27]. Flowering Chinese cabbage is now cultivated globally, particularly in Asian countries [26,28], and requires substantial nitrogen input to achieve optimal yield; hence, excessive NO_3_^−^ often accumulates in edible organs, especially in stalks, posing potential health risks [29,30]. Understanding the molecular mechanism of nitrogen uptake and transportation in flowering Chinese cabbage could inform strategies to optimize fertilization practices and improve NUE. Here, we present a genome-wide survey and detailed analysis of the *AMT* gene family in flowering Chinese cabbage. We examined their phylogenetic relationships, conserved motifs, gene structures, chromosomal localization, syntenic patterns, cis-regulatory elements, and expression patterns. Among these, *BcAMT1.1* showed strong transcriptional induction in a low-nitrogen atmosphere. Functional assays of *BcAMT1.1* in yeast mutant strain 31019b confirmed its functional NH_4_^+^ transport activity, and overexpression in *Arabidopsis* enhanced seedling growth, NH_4_^+^ uptake, and nitrogen assimilation-related gene transcription under low-nitrogen conditions. This study underscores the pivotal role of AMTs in plant growth and nitrogen metabolism, where BcAMT1.1 is critical for nitrogen acquisition and assimilation under low-nitrogen conditions. In addition, genetic resources can be provided to improve NUE in vegetable crops.

## 2. Results

### 2.1. Identification and Chromosomal Localization of the BcAMT Gene Family in Flowering Chinese Cabbage

**Figure 1:**
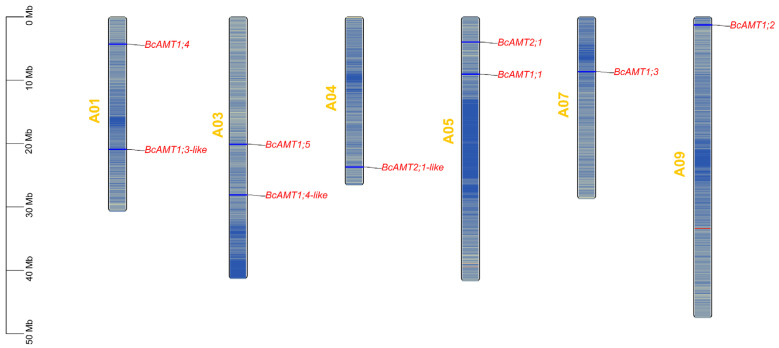
Chromosomal location of *BcAMT* genes in flowering Chinese cabbage. Bars represent individual chromosomes, with color gradients indicating gene density along each chromosome. Physical gene positions are marked, and the scale bar denotes the relative chromosome length in megabase (Mb) units.

Potential AMT members in the flowering Chinese cabbage genome were identified by conducting BLASTP searches (BLAST+ v2.16.0, NCBI, Bethesda, MD, USA) using *Arabidopsis* AMT protein sequences obtained from the BRAD. Candidate sequences were confirmed to contain the Pfam domain of the ammonium transporter family (PF00909). In total, nine BcAMT members were identified and designated BcAMT1.1 to BcAMT2.1-like based on their homology with *Arabidopsis* AMTs. The physicochemical properties of BcAMTs were assessed using the EXPASY ProtParam, including molecular weight (MW), isoelectric point (pI), number of amino acids, and grand average of hydropathy (GRAVY). The number of amino acids ranged from 476 (BcAMT1.3-like) to 512 aa (BcAMT1.2), with predicted MW ranging from 50.79 (BcAMT1.3-like) to 54.88 kDa (BcAMT1.2). The pI values varied from 5.45 (BcAMT1.4-like) to 7.73 (BcAMT1.2), indicating that most members were weakly acidic. All instability indices were below 40, and the GRAVY value was higher than zero, suggesting that these BcAMT proteins were stable and hydrophobic. Subcellular localization analysis identified all BcAMT proteins in the plasma membrane, with 9 to 11 transmembrane domains (Table 1), consistent with the membrane-associated transport functions of AMT. Chromosomal mapping showed that nine *BcAMTs* were dispersed across six chromosomes (Chr) of the flowering Chinese cabbage genome (Table 1; Figure 1); *BcAMT1.3-like* and *BcAMT1.4* were located on Chr1; *BcAMT1.4-like* and *BcAMT1.5* were located on Chr3; *BcAMT1.1* and *BcAMT2.1* were located on Chr5; and *BcAMT2.1-like*, *BcAMT1.3*, and *BcAMT1.2* were located on Chr4, Chr7, and Chr9.

### 2.2. Phylogenetic Tree and Conserved Domains Analyses of the BcAMT Gene Family

**Figure 2:**
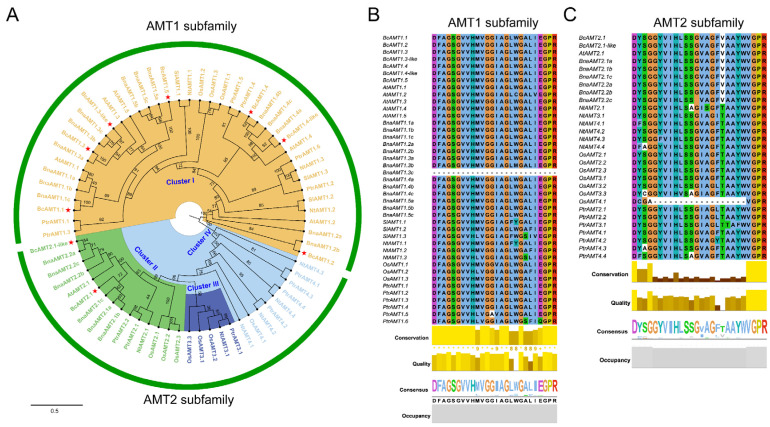
Phylogenetic relationships and conserved signature sequences of AMTs. (**A**) The AMT phylogenetic tree was created using MEGA 7.0, with bootstrap values calculated from 1000 replications. *At*: *Arabidopsis thaliana*; *Sl*: *Solanum lycopersicum*; *Nt*: *Nicotiana tabacum*; *Os*: *Oryza sativa*; *Ptr*: *Populus trichocarpa*; *Bn*: *Brassica napus*; *Bc*: *Brassica campestris*. The AMT members labeled in yellow belong to Cluster I, while those labeled in green, blue, and light blue correspond to Clusters II, III, and IV, respectively. BcAMTs are marked with a red pentalpha. (**B**) Conserved signature sequences of the AMT1 subfamily. (**C**) Conserved signature sequences of the AMT2 subfamily.

To evaluate the evolutionary relationships of AMT proteins, a maximum likelihood (ML) phylogenetic tree was generated in MEGA 7.0, incorporating AMTs from flowering Chinese cabbage, *Arabidopsis thaliana*, *Brassica napus*, *Solanum lycopersicum*, *Nicotiana tabacum*, *Oryza sativa*, and *Populus trichocarpa* (Table S1). The phylogenetic tree resolved into two dominant clades (Figure 2A): seven BcAMTs grouped within the AMT1 subfamily and two BcAMTs grouped within AMT2. Both AMT2 members were found in Cluster II, and no flowering Chinese cabbage or other Cruciferae AMTs were placed in Clusters III or IV. Homology analysis showed that BcAMTs shared high homology with *Arabidopsis* AtAMTs and rapeseed BnaAMTs, with orthologous counterparts found in flowering Chinese cabbage (Figure 2A). Furthermore, several paralogous gene pairs were identified, including *BcAMT1.3*/*BcAMT1.3-like*, *BcAMT1.4*/*BcAMT1.4-like*, and *BcAMT2.1*/*BcAMT2.1-like*, with each gene pair clustering with the corresponding AMT members in *Arabidopsis* and rapeseed, suggesting possible functional redundancy. Signature motif analysis, performed in Jalview v2.11.2.7, revealed that all AMT1 subfamily members shared the conserved sequence “DFAGSGVVHMVGGIAGLWGALIEGPR”, except BnaAMT1.3c in rapeseed; in addition, all AMT2 members universally contained the conserved sequence “DYSGGYIHLSSGVAGFTAAYW WGPR”, except OsAMT4.1 in *O. sativa* (Figure 2B,C). The signature motifs strongly support the phylogenetic classification.

### 2.3. Conserved Motifs and Gene Structure of the BcAMT Gene Family

**Figure 3:**
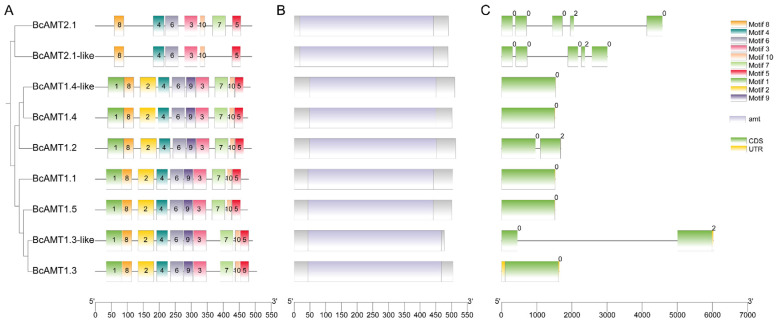
Conserved motif composition, domain architecture, and gene structure of BcAMTs. (**A**) Phylogenetic tree and distribution of motifs. A maximum likelihood (ML) phylogenetic tree of BcAMTs in flowering Chinese cabbage was generated in MEGA 7.0, and motif distributions were analyzed using MEME (https://meme-suite.org/meme/, accessed on 10 December 2025); numbers within different color boxes indicate distinct motifs identified in BcAMTs. (**B**) Conserved domain analysis. Light purple frames represent the conserved AMT domain in BcAMTs. (**C**) Gene structure analysis. Yellow boxes represent UTR regions, while green boxes and black lines indicate exons and introns; numbers above the boxes represent the phase value of each intron.

Motif composition and gene structure were analyzed in relation to evolutionary classification. Among all BcAMT members, ten consensus motifs were identified. There were ten motifs in the BcAMT1 subfamily, whereas BcAMT2.1 and BcAMT2.1-like contained seven and six motifs, respectively. A total of 6 conserved motifs (motifs 3, 4, 5, 6, 8, and 10) were conserved across all BcAMT members (Figure 3A). Domain prediction showed that all BcAMT proteins contained the characteristic ammonium transporter domain (amt) (Figure 3B). Gene structure analysis revealed marked differences between subfamilies: *BcAMT2* members harbored four introns, while most *BcAMT1* members were intronless, except for *BcAMT1.2* and *BcAMT1.3-like* (Figure 3C). These differences in gene structure may underpin functional divergence between BcAMT1 and BcAMT2 subfamilies.

### 2.4. Gene Duplication and Synteny Analyses of the BcAMT Gene Family

**Figure 4:**
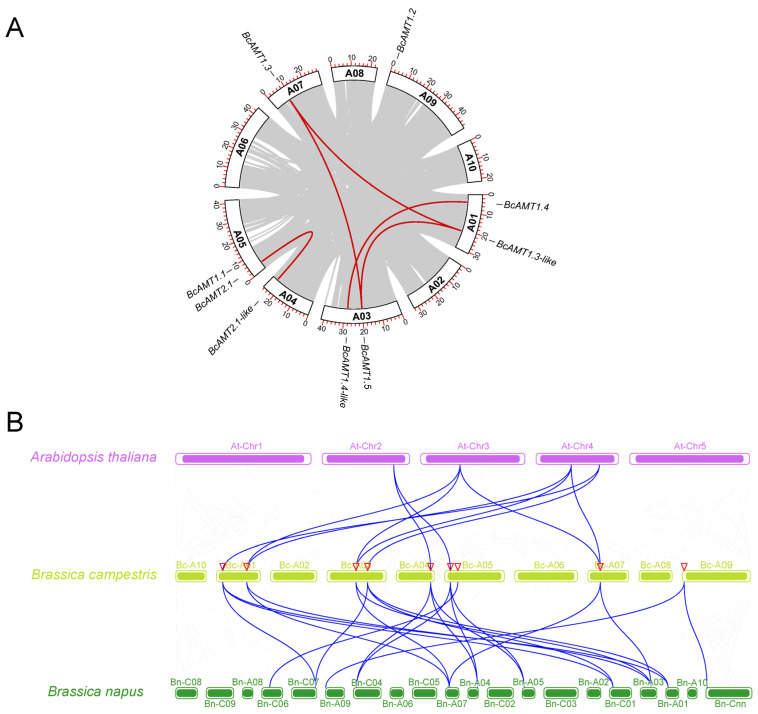
Collinearity patterns of the *AMT* gene family. (**A**) Collinearity relationships of *AMT*s in flowering Chinese cabbage. Grey boxes represent chromosomes of flowering Chinese cabbage, and red curves depict the paralogous gene pairs. (**B**) Comparative synteny among *A. thaliana*, *B. napus*, and flowering Chinese cabbage. Colored bars represent chromosomes, blue curves indicate the collinear gene pairs, and red triangles indicate AMTs in flowering Chinese cabbage.

To understand the evolutionary expansion of the *BcAMT* gene family, genome-wide collinearity analysis was performed using the MCScan X module in TBtools-II v2.345. Five segmentally gene pairs were identified: *BcAMT1.3*/*BcAMT1.3-like*, *BcAMT1.4*/*BcAMT1.4-like*, *BcAMT1.3*/*BcAMT1.5*, *BcAMT1.5*/*BcAMT1.3-like*, and *BcAMT2.1*/*BcAMT2.1-like* (Figure 4A; Table 2). Segmental duplications likely played a vital role in the expansion of the BcAMT family in flowering Chinese cabbage. Based on the rates of synonymous (Ks) and nonsynonymous (Ka) substitution, and assuming a neutral divergence rate of 1.5 × 10^−8^ substitutions per site each year, the divergence times of these paralogous pairs were estimated to range from 10.5503 to 24.7684 million years ago (MYA), averaging ~16.9060 MYA (Table 2). Ka/Ks ratios ranged from 0.0962 to 0.1383, remaining well below 1.0. This implies that *BcAMTs* duplicates have undergone intense selection processes throughout evolution.

Comparative synteny mapping revealed extensive collinear relationships between *BcAMT* genes and *AMT*s in *Arabidopsis* and *B. napus*; however, no syntenic associations were found with *O. sativa*. Seven *BcAMT* genes exhibited a syntenic correlation with *Arabidopsis AMT*s, and nine *BcAMT* genes correlated with *B. napus AMTs* (Figure 4B; Table S2). Specific relationships included *BcAMT1.3*, *BcAMT1.3-like*, and *BcAMT1.5* with *AtAMT1.1* and *AtAMT1.5*; *BcAMT1.4* and *BcAMT1.4-like* with *AtAMT1.4*; and *BcAMT2.1* and *BcAMT2.1-like* with *AtAMT2.1* (Figure 4; Table S2). In *B. napus*, many syntenic relationships were identified, including *BcAMT1.1* with *BnaAMT1.1b*, *BcAMT1.2* with *BnaAMT1.2a* and *BnaAMT1.2b*; *BcAMT1.3*, *BcAMT1.3-like* and *BcAMT1.5* with *BnaAMT1.3a* and *BnaAMT1.5a*; *BcAMT1.4* and *BcAMT1.4-like* with *BnaAMT1.4a*, *BnaAMT1.4b* and *BnaAMT1.4c*; and *BcAMT2.1* and *BcAMT2.1-like* with *BnaAMT2.1a*, *BnaAMT2.1c*, and *BnaAMT2.2c* (Figure 4; Table S2). These syntenic relationships highlight the conserved genomic context of *BcAMT*s within *Brassica* species and their divergence from monocot *AMTs*.

### 2.5. Analysis of Cis-Acting Elements in BcAMT Promoter Regions

**Figure 5:**
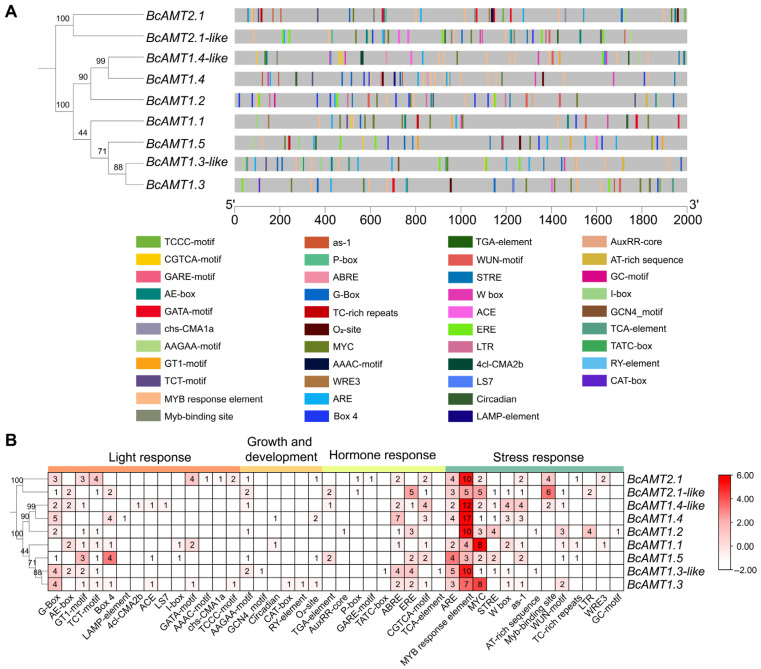
Analysis of *cis*-acting elements in *BcAMT* promoters. (**A**) Phylogenetic relationships and distribution of cis-acting elements. An ML phylogenetic tree of BcAMTs in flowering Chinese cabbage was generated in MEGA 7.0. Gray bars indicate the full length of *BcAMT1* promoters, and differently colored lines represent distinct cis-acting elements. (**B**) Number of each cis-acting element type. Different color boxes indicate element types as indicated in the legend, and numbers within the boxes represent the count of each element in *BcAMT* promoters.

To clarify transcriptional regulation of *BcAMT*, we analyzed *cis*-acting elements in 2000 bp upstream promoter regions using the PlantCARE database (https://bioinformatics.psb.ugent.be/webtools/plantcare/html/, accessed on 10 December 2025). In total, 42 types of elements were detected and grouped into four functional categories. (1) Light-responsive elements: 14 light-related regulatory motifs, including the G-Box, GT1-motif, and TCT-motif, were detected in most *BcAMT* promoters. (2) Growth- and development-related elements: the AAGAA-motif, O_2_-site, and CAT-box motifs were present in *BcAMT* promoters, while the circadian element was identified exclusively in the promoters of *BcAMT1.1* and *BcAMT1.4*. (3) Hormone-response elements: Promoters contained binding sites responsive to gibberellin (GARE-motif, TATC-box, P-box), auxin (AuxRR-core, TGA-element), abscisic acid (ABRE), salicylic acid (TCA-element), methyl jasmonate (CGTCA-motif), and ethylene (ERE). Notably, ABRE, ERE, and CGTCA-motif were widely distributed among promoters. (4) Stress-related elements: 13 types of stress-related motifs were detected, including stress-responsiveness element (STRE), anaerobic induction element (ARE), salicylic acid or pathogen-induced signaling (as-1), low-temperature responsiveness (LTR), wound-responsive element (WUN-motif), and several MYB/MYC-binding sites (Figure 5). These findings indicate that *BcAMT* transcription may be regulated by various environmental and endogenous factors, including light, hormonal signaling, growth and development, and abiotic or biotic stresses.

### 2.6. Expression Patterns of BcAMTs Across Tissues of Flowering Chinese Cabbage and Under Different Nitrogen Forms

#### 2.6.1. Tissue-Specific Expression

**Figure 6:**
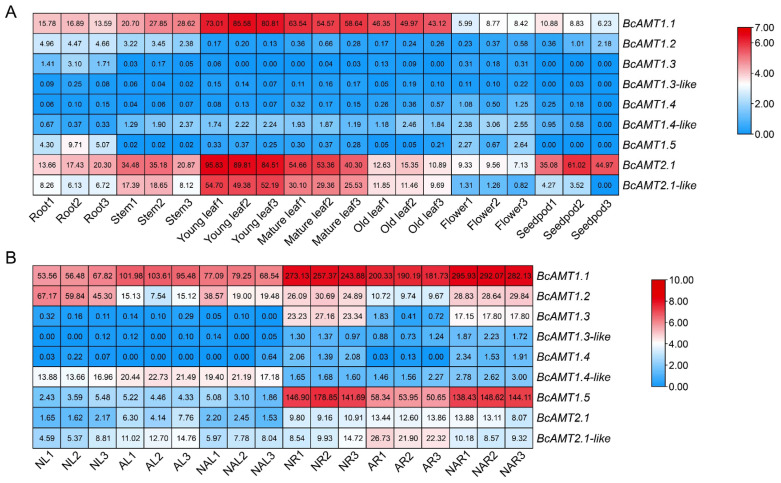
Expression characteristics of *BcAMTs* in flowering Chinese cabbage. (**A**) Expression in different tissues. (**B**) Expression under different nitrogen regimes. AL and AR: leaves and roots in 1 mmol·L^−1^ NH_4_^+^; NAL and NAR: leaves and roots in 0.5 mmol·L^−1^ NH_4_^+^ and 0.5 mmol·L^−1^ NO_3_^−^; NL and NR: leaves and roots in 1 mmol·L^−1^ NO_3_^−^. The heatmap shows normalized fragments per kilobase of exon model per million mapped reads (FPKM), and values in rectangles represent raw data.

RNA sequencing (RNA-seq) data from the cultivar “49 Caixin” (SRP427920) revealed distinct spatial expression patterns for *BcAMTs* in seven tissues (roots, stems, flowers, seedpods, and young/mature/senescent leaves). *BcAMT1.1*, *BcAMT2.1*, and *BcAMT2.1-like* were broadly expressed in tissues. Notably, *BcAMT1.1* showed high transcript abundance in leaves, stems, and roots, with fragments per kilobase of the exon model per million mapped reads (FPKM) exceeding 13; *BcAMT2.1* was highly expressed in young/mature leaves, seedpods, stems and roots (FPKM > 13); and *BcAMT2.1-like* was predominantly expressed in young and mature leaves and stems (FPKM > 8) (Figure 6A). In contrast, other *BcAMTs* exhibited relatively low expression levels (FPKM < 5) and tissue-specific expression patterns. *BcAMT1.2* was mainly expressed in the roots and stems; *BcAMT1.3* and *BAMT1.5* were root-specific; and *BcAMT1.4,* together with *BcAMT1.4-like,* was exclusively expressed in flowers. Among them, the expression of *BcAMT1.3-like* was nearly undetectable across all examined tissues (Figure 6A). The results indicate that *BcAMTs* may play distinct roles in regulating plant growth and development in flowering Chinese cabbage.

#### 2.6.2. BcAMT Response to Nitrogen Forms

Changes in expression were evaluated following treatments with different nitrogen sources: 1 mmol·L^−1^ NH_4_^+^, 0.5 mmol·L^−1^ NH_4_^+,^ and 0.5 mmol·L^−1^ NO_3_^−^, and 1 mmol·L^−1^ NO_3_^−^ for 4 d (PRJCA021671). The transcripts of *BcAMT1.1* were abundant in both leaves and roots in response to different nitrogen sources. In leaves, *BcAMT1.1* transcription was significantly upregulated under both NH_4_^+^ and mixed nitrogen nutrition compared to NO_3_^−^ treatment; in roots, *BcAMT1.1* expression was reduced by NH_4_^+^ but increased under the mixed treatment (Figure 6B). The transcription of *BcAMT1.3* and *BcAMT1.5* in leaves was unaffected by nitrogen; in the roots, they were significantly decreased by NH_4_^+^ and only slightly altered by the mixed nitrogen treatment (Figure 6B). In contrast, the transcripts of *BcAMT2.1* and *BcAMT2.1-like* were strongly induced by NH_4_^+^ and the mixed nitrogen nutrition in both roots and leaves, compared with *BcAMT1.1* exposed to NO_3_^−^ (Figure 6B). These results show that *BcAMTs* exhibited dynamic responses to various nitrogen treatments, suggesting their potential role in nitrogen uptake and metabolism. Despite several *BcAMT1s* being significantly regulated by nitrogen treatments, we selected BcAMT1.1 for further functional characterization due to its highest absolute expression in tissue-specific analyses and significant response to nitrogen availability, particularly in the roots.

#### 2.6.3. Response to Nitrogen Deficiency and Different NH_4_^+^ Concentrations

**Figure 7:**
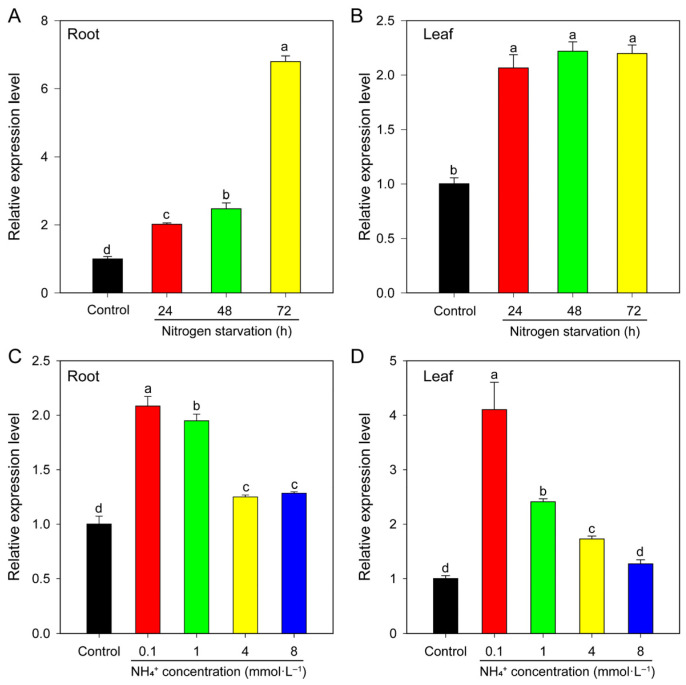
*BcAMT1.1* expression patterns in flowering Chinese cabbage determined by qRT-PCR under different nitrogen regimes. After cultivation for two weeks at 4 mmol·L^−1^ NO_3_^−^, seedlings of flowering Chinese cabbage were subjected to different nitrogen treatments. (**A**,**B**) *BcAMT1.1* expression in roots and leaves under nitrogen deficiency for 0, 24, 48, and 72 h. (**C**,**D**) *BcAMT1.1* expression in roots and leaves subjected to different NH_4_^+^ concentrations for 2 h. The data represent the mean ± standard deviation (SD) (n = 3). Different lowercase letters present the differences at the 0.05 level.

The quantitative real-time PCR (qRT-PCR) analysis revealed that the *BcAMT1.1* transcript was markedly induced under nitrogen deficiency. After 72 h of nitrogen starvation, *BcAMT1.1* expression increased 6.78-fold in roots and 2.01-fold in leaves compared to the control (Figure 7A,B). When exposed to various NH_4_^+^ concentrations, *BcAMT1.1* transcripts were significantly upregulated with lower NH_4_^+^ levels, reaching 1.95~4.10 times that of the control. In contrast, *BcAMT1.1* was clearly downregulated by higher NH_4_^+^ concentrations, which were only 1.25~1.73 times compared to the control (Figure 7C,D). Considering the established role of AMT1 subfamily members in high-affinity NH_4_^+^ uptake, and their inducibility under nitrogen deficiency or low-nitrogen conditions, *BcAMT1.1* was selected for subsequent functional characterization.

### 2.7. Subcellular Localization and NH_4_^+^ Transport Activity of BcAMT1.1

**Figure 8:**
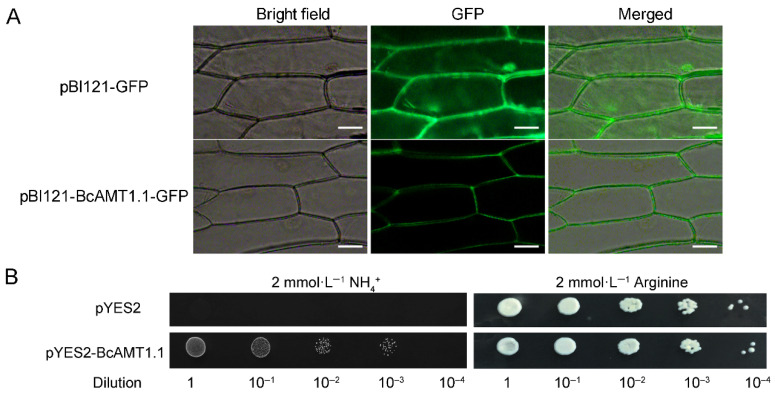
Subcellular localization and NH_4_^+^ transport activity of BcAMT1.1. (**A**) Subcellular localization of BcAMT1.1. Scale bar is 50 μm. (**B**) Yeast complementation assay of BcAMT1.1 in mutant strain 31019b.

Transient expression assays indicated that BcAMT1.1 was localized in the plasma membrane of onion epidermal cells (Figure 8A). We further explored the role of BcAMT1.1 plays in NH_4_^+^ transport using the yeast mutant 31019b. Transformants harboring pYES2-BcAMT1.1 grew normally on a medium supplied with 2 mmol·L^−1^ NH_4_^+^ (Figure 8B). These findings indicate that BcAMT1.1 can complement the growth defect of the mutant, confirming its potential involvement in ammonium transport and utilization in yeast.

### 2.8. Overexpressing BcAMT1.1 Promotes NH_4_^+^ Uptake and Accelerates Plant Growth of Arabidopsis Under Low-NH_4_^+^ Concentrations

**Figure 9:**
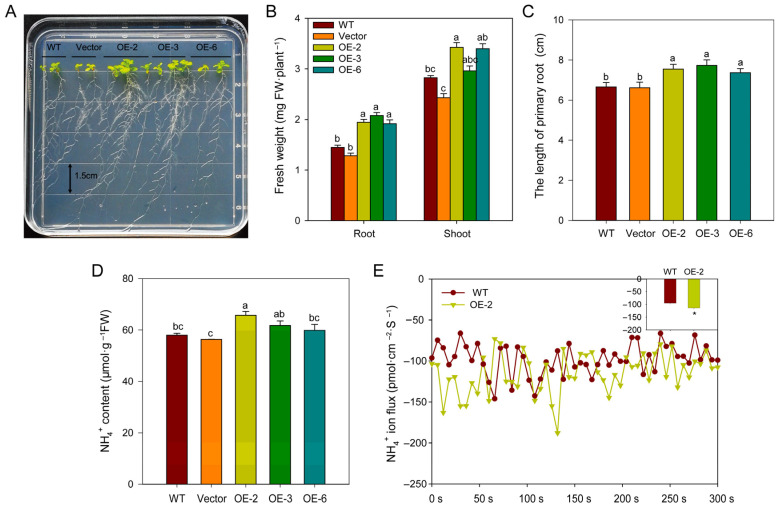
Phenotype and physiological traits of *BcAMT1.1*-overexpressing *Arabidopsis* under 0.25 mmol·L^−1^ NH_4_^+^. (**A**) Growth phenotype of wildtype (WT), alongside vector control and lines overexpressing *BcAMT1.1*. After preculturing on a solid medium containing 4 mmol·L^−1^ NO_3_^−^ for 4 d, seedlings were transferred to 0.25 mmol·L^−1^ NH_4_^+^ for 10 d. (**B**) Fresh weight of whole pants. (**C**) Primary root length. (**D**) NH_4_^+^ content of whole plants. (**E**) NH_4_^+^ ion flux for the root surface. WT: wildtype; vector: overexpressing pCAMBIA 3301 vector; OE-2, OE-3, and OE-6: overexpression lines 2, 3, and 6. The line chart shows the real-time change in NH_4_^+^ ions for the root’s surface over 5 min; a small histogram presents the net NH_4_^+^ change. The data represent the mean ± SD (n = 10 in **B**,**C**, n = 3 in **D**, and n = 6 in **E**). Lowercase letters or asterisks denote significant differences (*p* < 0.05).

To evaluate the potential role of BcAMT1.1, three independent T_4_ homozygous *Arabidopsis* lines were selected for analysis (Figure S1). After pre-cultivation on a 4 mmol·L^−1^ NO_3_^−^ medium for 4 d, seedlings were subsequently transferred to 0.25 mmol·L^−1^ NH_4_^+^ vertical agar plates for 10 d. Compared with wildtype (WT), overexpressing *BcAMT1.1* clearly promoted the growth of *Arabidopsis* seedlings, with the fresh weight of shoots and roots increasing by 1.32~1.34 times and 2.04~2.36 times, respectively. The primary root length was increased by 1.10~1.16-fold (Figure 9A–C), and NH_4_^+^ content in transgenic lines was 1.03~1.13 times higher than WT (Figure 9D). The net NH_4_^+^ influx rate was measured in the OE-2 line, and increased by 20% compared to WT (Figure 9E).

The physiological relevance of this trait was confirmed by sensitivity testing with methylammonium (MeA), which is a toxic analog of NH_4_^+^. For the 20 mmol·L^−1^ MeA medium, overexpression lines showed severe growth inhibition, with fresh weight reduced by 35~48% WT, accompanied by chlorosis and shorter primary roots (Figure S2). Collectively, these results demonstrate that overexpressing *BcAMT1.1* enhances NH_4_^+^ uptake capacity under low-NH_4_^+^ conditions and promotes plant growth.

### 2.9. Overexpressing BcAMT1.1 Alters Nitrogen Ion Fluxes and the Expression of Nitrogen Assimilation-Related Genes in Arabidopsis

**Figure 10:**
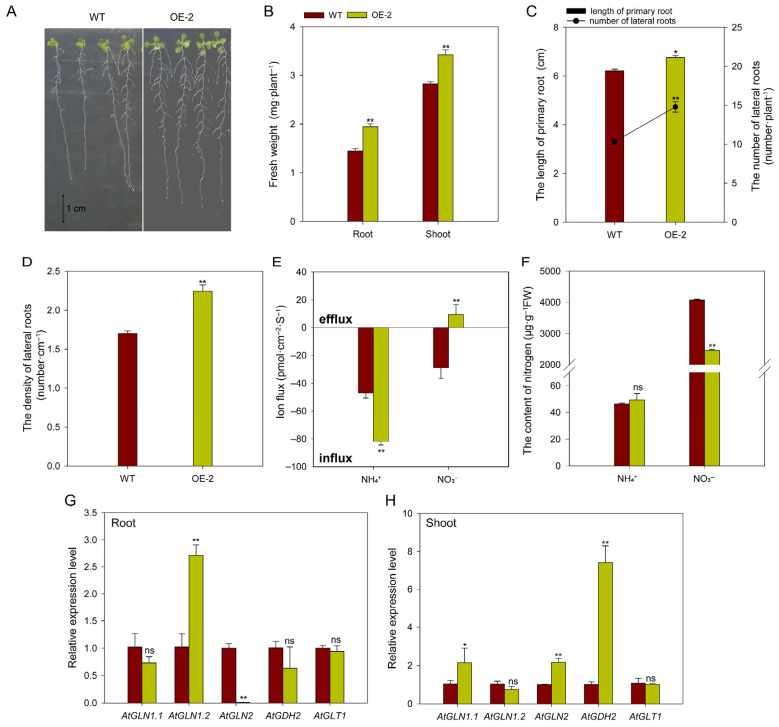
Effect of overexpressing *BcAMT1.1* on plant growth, nitrogen uptake, and assimilation under mixed NH_4_^+^ and NO_3_^−^ nutrition. (**A**) Plant phenotype. (**B**) Fresh weight of roots and shoots. (**C**) Primary root length and lateral root numbers. (**D**) Lateral root density. (**E**) NH_4_^+^ and NO_3_^−^ ion fluxes. (**F**) NH_4_^+^ and NO_3_^−^ content. (**G**,**H**) Expression of nitrogen assimilation-related genes in roots and shoots. The data represent the mean ± SD (n = 10 in **B**–**D**; n = 6 in **E**; and n = 3 in **F**–**H**). WT: wildtype; OE-2: overexpression line 2. ns: no significant difference; * or ** represents significant differences at *p* < 0.05 or *p* < 0.01, respectively.

To further explore the function of BcAMT1.1, the OE-2 line was used under mixed nitrogen conditions (0.0625 mmol·L^−1^ NH_4_^+^ and 0.1875 mmol·L^−1^ NO_3_^−^). Overexpressing *BcAMT1.1* lines exhibited better growth potential than WT, characterized by significantly increased biomass, elongated primary root length, and increased number and density of lateral roots (Figure 10A–D). Net NH_4_^+^ influx in OE-2 was 1.74-fold higher than in WT, whereas the NO_3_^−^ flux shifted from net influx in WT to net efflux in OE-2 (Figure 10E). The expression levels of *AtNRT1.1* and *AtNRT2.1* were significantly decreased in the roots of OE-2, at 18.72% and 22.31% of WT plants, respectively (Figure S3). The NO_3_^−^ content significantly decreased in transgenic seedlings, whereas the NH_4_^+^ content remained unchanged (Figure 10F).

Furthermore, we detected transcript levels of key genes related to nitrogen assimilation, including *GLN*, *GLT*, and *GDH*, and encoding GS, GOGAT, and glutamate dehydrogenase. Transcript analysis of nitrogen assimilation-related genes revealed that overexpressing *BcAMT1.1* significantly upregulated *AtGLN1.2* transcription to about 2.60-fold that of WT, and significantly downregulated *AtGLN2* expression in the roots. It exerted no clear influence on *AtGLN1.1*, *AtGDH2*, or *AtGLT1* (Figure 10G). In the shoots, the transcription of *AtGLN1.1*, *AtGLN2*, and *AtGDH2* in overexpression lines was significantly improved by 2.08, 2.16, and 7.31 times, respectively, compared to WT (Figure 10H).

### 2.10. Protein–Protein Interaction (PPI) Network of BcAMT1.1

**Figure 11:**
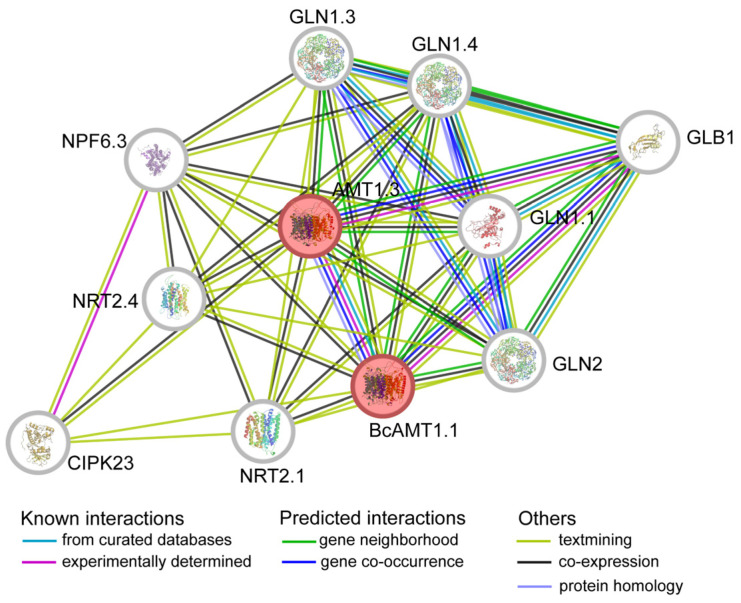
Predicted protein–protein interaction network of BcAMT1.1. AMT1.1/1.3: ammonium transporter 1.1/1.3; NRT2.1/2.4: nitrate transporter 2.1/2.4; NPF6.3: nitrate transporter 1/peptide transporter family 6.3; GLN1.1/1.3/1.4/2: glutamate synthetase 1.1/1.3/1.4/2; GLB1: PII nitrogen sensing protein; and CIPK23: CBL-interacting protein kinase 23.

To further elucidate the potential molecular mechanism of BcAMT1.1, a PPI network was inferred from orthologous genes in *Arabidopsis* using the STRING database. The analysis identified 46 putative interaction pairs (Figure 11; Table S3). In this network, BcAMT1.1 was predicted to interact with AMT1.3, a nitrate transporter (NPF6.3/NRT1.1, NRT2.1, and NRT2.4), GLN (GLN1.1, GLN1.3, GLN1.4, and GLN2), and GLB1, which is a PII protein involved in the nitrogen-sensing signal transduction pathway [31]. In addition, BcAMT1.1 may interact with CBL-interacting protein kinase 23 (CIPK23), which forms a complex with the calcineurin B-like protein (CBL). This plays a prominent role in activating the plant nutrient transporter [32]. Notably, CIPK23 was also predicted to interact with AMT1.3, NRT2.1, NRT2.4, and NPF6.3. The observations suggest that AMT1.1 may participate in nitrogen absorption and assimilation by interacting with CIPK23 and other key regulators within the nitrogen metabolism network.

## 3. Discussion

AMT proteins are key mediators of NH_4_^+^ absorption and its transport from the rhizosphere to the intercellular space, thereby maintaining cellular NH_4_^+^ homeostasis [14,33]. Although the *AMT* gene families have been well characterized in a variety of species, such as *Arabidopsis*, *O. sativa*, and *B. napus*, a comprehensive and systematic identification in flowering Chinese cabbage remains lacking. In this study, nine *BcAMT* genes were systematically identified and categorized into two phylogenetic subfamilies: AMT1 (7 members) and AMT2 (2 members), which is consistent with the classification of AtAMTs in *Arabidopsis* and BnaAMTs in *B. napus* (Figure 2). Notably, while each *Arabidopsis* AMT has a single homologous copy, most *B. napus* AMTs occur as 2~3 copies. In flowering Chinese cabbage, BcAMT1.1, BcAMT1.2, and BcAMT1.5 contain single copies, while BcAMT1.3, BcAMT1.4, and BcAMT2.1 contain two copies. These homologous genes clustered into corresponding branches with AMT proteins from other species support previous evidence (Figure 2). Consistent with the higher number of duplicated orthologs in *Brassica* species compared to *Arabidopsis* [34], higher levels of duplicated orthologs were found in *Brassica* compared to *Arabidopsis*. A previous study reported on three polyploidization events in *B. rapa*: γ triplication (135 MYA), β duplication (90~100 MYA), and α duplication (24~40 MYA) [35]. In our study, five out of nine *BcAMT* genes (55.56%) were products of segmental duplication (Figure 4; Table 2), suggesting that the expansion of *BcAMTs* in flowering Chinese cabbage was primarily driven by segmental duplication.

Polyploidization often leads to diversification in both structural features and functional domains of genes. In this study, motif analysis revealed that BcAMT1 subfamily members in flowering Chinese cabbage possess 10 uniform motifs, whereas BcAMT2 members lack motifs 1, 2, and 9, with BcAMT2.1-like further lacking motif 7 (Figure 3). Similar reductions in conserved motif numbers were reported in the AMT2 subfamily compared with AMT1 in *B. napus* [19]. Previous studies reported that most *AMT1* genes lack introns, with exceptions including *LjAMT1.1* in *Lotus japonicus* [36], *SlAMT1.2* in *Solanum lycopersicum* [37], and *MeAMT1.2* in *Manihot esculenta* [38]. In this study, both *BcAMT1.2* and *BcAMT1.3-like* contained one intron, and the intron length of *BcAMT1.3-like* exceeded 4500 bp (Figure 3). A similar long intron structure was found in rapeseed *BnaAMT1.3a* [19]. All *BcAMT2* genes contained four introns and five exons, which is consistent with those reported in *Arabidopsis* [33], *B. napus* [19], and soybean [20]. Previous studies showed that all AMT members from *Chlamydomonas* are clustered in the AMT1 subfamily, with no AMT2-type members detected [39]. This observation suggests that these AMT2 genes in higher plants may be subject to more complex regulation, i.e., chromatin assembly, mRNA transport, and alternative splicing [40].

In plants, members of the AMT1 subfamily are widely recognized as high-affinity ammonium transporters [11,22], whereas AMT2 members are primarily associated with low-affinity transport [33]. In flowering Chinese cabbage, *BcAMTs* exhibit distinct expression profiles, and *BcAMT1.5* is predominantly expressed in the roots [41]. In this study, RNA-seq analysis revealed that *BcAMT1.1*, *BcAMT2.1*, and *BcAMT2.1-like* were mainly expressed in the roots, stems, and leaves. Notably, *BcAMT1.1* was strongly induced under nitrogen-deficient or low-nitrogen availability, particularly in the roots (Figure 7). This is consistent with previous studies where *AtAMT1.1* in *Arabidopsis* [42], *OsAMT1.1* in *O. sativa* [18], and *PsAMT1.1* in *Populus simonii* [43] were found to be induced under limited nitrogen availability and function as high-affinity ammonium transporters.

In the present study, BcAMT1.1 was located at the plasma membrane and was able to complement the growth defect of the yeast mutant 31019b on a medium containing 2 mmol·L^−1^ NH_4_^+^. In *Arabidopsis*, overexpressing *BcAMT1.1* significantly promoted plant growth in low-nitrogen conditions, which increased both net NH_4_^+^ influx and NH_4_^+^ content in comparison with WT. This indicates that *BcAMT1.1* encodes a high-affinity ammonium transporter in flowering Chinese cabbage, and is consistent with previous reports of AMT1.1 in *Arabidopsis* [22,42], *Oryza sativa* [18,44], and *P. simonii* [43], as well as the results of BcAMT1.2 and BcAMT1.5 in *B. campestris* [41,45]. This suggests that AMT1 members primarily function in NH_4_^+^ absorption, and their overexpression lines can enhance NH_4_^+^ absorption at low-NH_4_^+^ conditions. Under the mixed nitrogen nutrition, *BcAMT1.1*-overexpressing lines enhanced the transcription of *AtGLN1.1*, *AtGLN1.2*, *AtGLN2*, and *AtGDH2*, which are the key genes of GS/GOGAT or the GDH pathway, leading to increased net NH_4_^+^ influx without significant changes in NH_4_^+^ content in comparison to WT. This is in accordance with our earlier observations, which showed that overexpressing *BcAMT1.2* or *BcAMT1.5* significantly enhanced the transcription of most nitrogen assimilation-related genes [41,45]. A previous study also indicated that potential NH_4_^+^ toxicity can be mitigated if nitrogen assimilation rates match NH_4_^+^ uptake [46].

Beyond their role in NH_4_^+^ absorption, AMTs may also affect NO_3_^−^ uptake, as the acquisition of these two nitrogen sources can be synergistically regulated [4]. In *BcAMT1.1*-overexpressing lines, the transcript levels of *NRT1.1* and *NRT2.1*, which are responsible for NO_3_^−^ uptake in the roots [47], were significantly downregulated (Figure S3). Consistently, the net NO_3_^−^ flux shifted from influx in WT to efflux in *BcAMT1.1*-overexpressing lines, resulting in a marked decrease in NO_3_^−^ concentration. This pattern is similar to that observed for *BcAMT1.2* in flowering Chinese cabbage [45], while contrasting with the results for *PsAMT1.1* in *P. simonii* [43] and *BcAMT1.5* in flowering Chinese cabbage [41]. This suggests that AMT members may have divergent functions in modulating the interaction between NH_4_^+^ and NO_3_^−^ uptake [45]. NO_3_^−^ is reduced to nitrate (NO_2_^−^) by nitrate reductase (NR), and NO_2_^−^ needs to be rapidly converted to NH_4_^+^ to avoid toxicity. NH_4_^+^, as a metabolic product, can inhibit the feedback of NO_2_^−^ reduction, thereby increasing the risk of NO_2_^−^ accumulation [4,47]. When NH_4_^+^ and NO_3_^−^ coexist, NH_4_^+^ may trigger NO_3_^−^ efflux to prevent the accumulation of NO_2_^−^, thereby limiting further NO_3_^−^ uptake and subsequent NO_2_^−^ generation. It implies that AMTs could modulate nitrogen acquisition not only by mediating NH_4_^+^ transport, but also by regulating NR-driven NO_3_^−^ or NO_2_^−^ reduction processes.

AMT activities are strictly regulated to adapt to external nitrogen status at both the transcriptional and post-transcriptional levels [48,49,50]. The uptake of nutrients, such as NO_3_^−^ and NH_4_^+^, is often modulated by protein kinase-mediated phosphorylation at post-transcriptional levels [51]. In green algae, mitogen-activated protein kinase (MAPK) cascades, including MAPKKKs, MAPKKs, and MAPKs, modulate nitrogen assimilation by phosphorylating nitrate reductase (NR) to promote nitric oxide (NO) production; MAPKKKs RAF14 and RAF79 may play critical roles in nitrogen metabolism [52]. In higher plants, the protein kinase CIPK23 regulates NO_3_^−^ uptake by phosphorylating a threonine in NPF6.3/NRT1.1 [32,53,54]. Similarly, conserved threonine residues in AtAMT1.1 and AtAMT1.2 are targeted for phosphorylation by the CBL1-CIPK23 complex [32,49,51]. In *Arabidopsis*, phosphorylation of threonine residues T464 and T494 in AtAMT1.3 serves as a key regulatory switch, enabling plants to fine-tune their response to varying nitrogen forms [50]. In the present study, the PPI network indicated that BcAMT1.1 might interact with CIPK23, as well as other proteins involved in nitrogen uptake and metabolism, including AMT, NRT, GLN, and GLB proteins (Figure 11). These findings suggest that CIPK23 might contribute to regulating nitrogen uptake and assimilation by modifying phosphorylation residues on AMT, NRT, or other interacting proteins. It is crucial to further identify novel upstream regulators of AMT1s to elucidate the molecular mechanisms of nitrogen transport [50].

## 4. Materials and Methods

### 4.1. Genome-Wide Identification of AMT Genes in Flowering Chinese Cabbage and Chromosome Location

The genome database of flowering Chinese cabbage was obtained from the China National Gene Bank (CNGB), published by Li et al. [34]. Sequences of AtAMT in *Arabidopsis* were acquired from the *Brassicaceae* Database (BRAD) (http://brassicadb.cn/, accessed on 12 July 2024) and utilized as query inputs for BLASTP analysis against the flowering Chinese cabbage genome (E-value < 1 × 10^−5^) using BLAST+. To confirm domain composition, candidate sequences were analyzed in the Pfam database (https://pfam.xfam.org/, accessed on 12 July 2024) to identify the presence of the ammonium transporter family domain (Pfam ID: PF00909). Physicochemical properties of predicted AMT proteins were estimated using the ExPASY ProtParam (https://web.expasy.org/protparam/, accessed on 12 July 2024), and conserved domains were examined using the NCBI Batch CD-Search tool (https://www.ncbi.nlm.nih.gov/Structure/bwrpsb/bwrpsb.cgi, accessed on 12 July 2024). Nine AMT proteins in flowering Chinese cabbage were finally confirmed as AMT family members. Predictions of subcellular localization were conducted with Deeploc-2.0 (https://services.healthtech.dtu.dk/cgi-bin/webface2.cgi, accessed on 12 July 2024), while transmembrane helices were identified using TMHMM v2.0 (https://services.healthtech.dtu.dk/services/TMHMM-2.0/, accessed on 12 July 2024). Chromosomal localization of *AMT* genes was analyzed and visualized using TBtools-II v2.345 [55].

### 4.2. Phylogenetic Tree, Conserved Motifs, Domains, and Gene Structure Analyses of AMT Members

Homologous AMT protein sequences of *A. thaliana*, *B. napus*, *S. lycopersicum*, *N. tabacum*, *O. sativa*, and *P. trichocarpa* were obtained from a public database (BRAD, GenBank, UniProt, Genoscope) and the literature [18,19,21,22]. A maximum-likelihood (ML) phylogenetic tree was generated based on the multiple sequence alignment of these seven species with 1000 bootstrap replicates, and was visualized using Figtree v1.4.4. Sequences alignments were visualized, and subfamily-specific conserved signature sequences were identified using Jalview v2.11.2.7 [56]. Conserved motifs of flowering Chinese cabbage AMTs were performed using MEME Suite (https://meme-suite.org/meme/, assessed on 20 July 2024), followed by analysis and visualization of domain organization and gene structures with TBtools-II.

### 4.3. Gene Duplication and Genome-Wide Synteny Analysis of AMTs

Gene duplication events were analyzed, and Ks and Ka parameters were calculated utilizing the MCScan X module in TBtools-II. The divergence rate (λ) was set to 1.5 × 10^−8^ for *B. rapa* [57]; divergence time (T) was estimated as T = Ks/2λ; and the Ka/Ks ratio was calculated. Comparative synteny between flowering Chinese cabbage, *A. thaliana*, *B. napus*, and *O. sativa* was evaluated and visualized in TBtools-II.

### 4.4. Identification of Cis-Acting Elements in AMT Promoter Regions

The 2000 bp upstream sequences of *AMT* genes were obtained from the flowering Chinese cabbage genome via TBtools-II, and cis-acting regulatory elements were identified using the PlantCARE database (https://bioinformatics.psb.ugent.be/webtools/plantcare/html/, assessed on 23 August 2024). Elements were classified according to their functional categories, and distribution patterns were visualized using TBtools-II.

### 4.5. Expression Profiling of BcAMTs

Expression levels of *BcAMT*s in different tissues (roots, stems, young leaves, mature leaves, old leaves, flowers, and seedpods) were retrieved from RNA-seq data (SRP427920) of the flowering Chinese cabbage cultivar “49 Caixin”. Expression patterns under different nitrogen forms were obtained from unpublished RNA-seq datasets (PRJCA021671) of cultivar “Youlv 501”. After 4 d of nitrogen starvation, seedlings were treated with 1 mmol·L^−1^ NH_4_^+^, 0.5 mmol·L^−1^ NH_4_^+^ and 0.5 mmol·L^−1^ NO_3_^−^, and 1 mmol·L^−1^ NO_3_^−^ for 4 d, the roots and leaves of which were collected for RNA sequencing. The values of FPKM were utilized to generate heatmaps with TBtools-II.

The experiments were performed in a controlled growth chamber, with conditions set to 23 ± 2 °C, 70% relative humidity, a 16 h light/8 h dark photoperiod, and a photosynthetic photon flux density of 150 μmol·m^−2^·s^−1^. Seedlings of the flowering Chinese cabbage cultivar “Youlv 501” were grown to the three-leaf stage in a modified Hoagland solution with 4 mmol·L^−1^ NO_3_^−^ for two weeks. Seedlings were rinsed thoroughly with deionized water and transplanted to a nitrogen-free-modified Hoagland solution for nitrogen starvation. Leaves and roots were collected at 0, 24, 48, and 72 h after the initiation of nitrogen deprivation. The remaining seedlings were subsequently exposed to NH_4_^+^ at concentrations of 0.1, 1, 4, and 8 mmol·L^−1^ for 2 h, after which the roots and leaves were harvested. For each treatment, three independent biological replicates were conducted, with each replicate consisting of samples collected from four different seedlings. Total RNA extraction, first-strand cDNA synthesis, and qRT-PCR were conducted with *GAPDH* used as the internal reference, as described by Zhu et al. [41].

### 4.6. Subcellular Localization of BcAMT1.1

Using the primers listed in Table S4, the coding sequence (CDS) of *BcAMT1.1* was amplified and cloned without the termination codon into the pBI121-GFP vector after it was linearized by *Sma* I and *Xba* I. The BcAMT1.1-GFP plasmid was transformed into onion epidermal cells via *Agrobacterium tumefaciens* EHA105. GFP signals were observed using a Zeiss Axio Imager D2 fluorescence microscope (Zeiss, Dresden, Germany).

### 4.7. Functional Complementation Analysis of BcAMT1.1 in Yeast

Using the ClonExpress II OneStep Cloning Kit (Vazyme Biotech, Nanjing, China), the *BcAMT1.1* CDS was inserted into the pYES2 vector and digested by *EcoR* I and *Xba* I. Recombinant plasmid and empty vector controls were transformed into yeast mutant strain 31019b (*Δmep1*, *Δmep2*, *Δmep3*, and *ura3*) via the lithium acetate method. This strain is unable to grow on a medium containing NH_4_^+^ concentrations below 5 mmol·L^−1^ as the sole nitrogen source [22]. Transformants were cultured at 30 °C for 3 d on a yeast nitrogen base medium (2% galactose) containing 2 mmol·L^−1^ arginine or NH_4_^+^ as the sole nitrogen source.

### 4.8. Overexpression of BcAMT1.1 in Arabidopsis

*BcAMT1.1* CDS was cloned into pCAMBIA3301 and transformed into *Arabidopsis* via a floral dip using *Agrobacterium tumefaciens* GV1301 [58]. Transgenic seeds were screened on phosphinothricin and analyzed by qRT-PCR. T_4_ generation homozygous lines were used to analyze plant phenotype and physicochemical indices.

Sterilized seeds were germinated on a 1/2 Murashige and Skong (MS) agar medium containing 4 mmol·L^−1^ NO_3_^−^ for 4 d, and then transferred to 1/2 MS plates with 0.25 mmol·L^−1^ NH_4_^+^ for 10 d. Fresh weight, primary root length, and NH_4_^+^ content were measured as described by Zhu et al. [45]. For each line, ten seedlings were used to measure fresh weight and primary root length, while fifteen seedlings were divided into three biological replicates for the determination of NH_4_^+^ content.

For mixed nitrogen treatments, *Arabidopsis* seedlings were pre-cultured on 1/2 MS medium supplemented with 4 mmol·L^−1^ NO_3_^−^ for 7 d, followed by a transfer to 1/2 MS medium with 0.0625 mmol·L^−1^ NH_4_^+^ and 0.1875 mmol·L^−1^ NO_3_^−^ for another 7 d. NH_4_^+^ and NO_3_^−^ contents were determined following the method of Ivančič and Degobbis [59]. Gene expressions were analyzed via qRT-PCR using TB Green^®^ Premix Ex Taq^TM^ II (TaKaRa Bio, Shiga, Japan), with *ACTIN2* as the internal reference. Primer sequences are listed in Table S4. Relative transcript levels were evaluated using the 2^−ΔΔCT^ method [60].

For ion flux assays, *Arabidopsis* seeds (WT and OE-2 line) were pre-cultured on 1/2 MS medium supplemented with 4 mmol·L^−1^ NO_3_^−^ for 4 d and then subjected to a nitrogen-free 1/2 MS medium for 7 d. The seedlings were subsequently placed in the measuring solution containing 0.25 mmol·L^−1^ NH_4_^+^ (or 0.0625 mmol·L^−1^ NH_4_^+^ + 0.1875 mmol·L^−1^ NO_3_^−^), 0.1 mmol·L^−1^ CaCl_2_, and 0.3 mmol·L^−1^ MES to determine ion fluxes using the scanning ion-selective electrode technique, according to the method described by Zhu et al. [45]. Six uniform seedlings were used for ion flux measurements in each treatment.

### 4.9. Prediction of Protein–Protein Interaction Network of BcAMT1.1

A potential protein interaction partner of BcAMT1.1 was predicted using the STRING database (https://cn.string-db.org, accessed on 25 August 2024), with *Arabidopsis* orthologs used as the reference.

### 4.10. Statistical Analysis

Statistical analyses were conducted using SPSS v21.0 (IBM, Armonk, NY, USA). Differences among treatments were assessed using one-way ANOVA followed by Duncan’s multiple range test, with significance defined at *p* < 0.05 or *p* < 0.01. The data represent the mean ± standard deviation (SD) (n = 3~10). In the figures, distinct lowercase letters or asterisks denote statistically significant differences among treatments.

## 5. Conclusions

**Figure 12:**
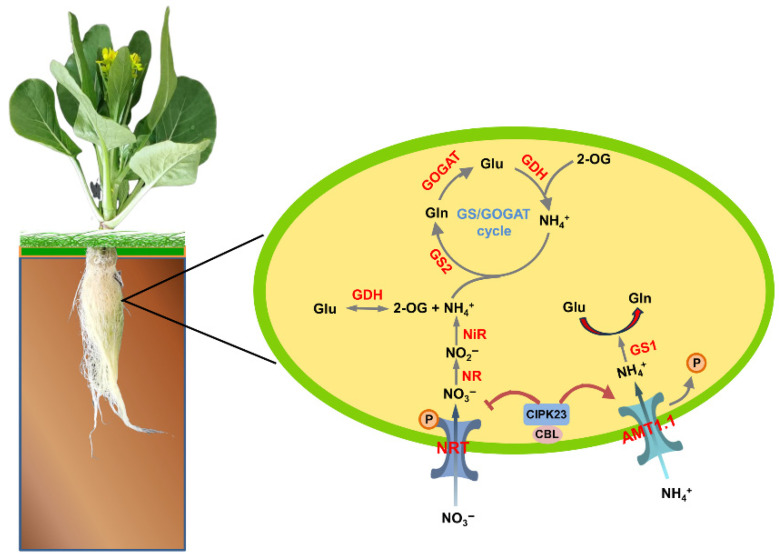
A proposed model for BcAMT1.1 regulating nitrogen uptake and assimilation under low-nitrogen conditions. When NH_4_^+^ and NO_3_^−^ coexist, AMT1.1 facilitates high-affinity NH_4_^+^ uptake into the root cells, where it is incorporated into glutamine (Gln) via glutamine synthetase 1 (GS1) and further processed in the GS/GOGAT cycle to produce glutamate (Glu). Glutamate dehydrogenase (GDH) also contributes to NH_4_^+^ assimilation. NO_3_^−^ is absorbed via nitrate transporters (NRTs), reduced to nitrite (NO_2_^−^) by nitrate reductase (NR), and then further reduced to NH_4_^+^ by nitrite reductase (NiR). The calcineurin B-like protein (CBL)-interacting protein kinase 23 (CIPK23) and CBL complex may potentially contribute to the coordination of NH_4_^+^ and NO_3_^−^ uptake or assimilation via phosphorylation.

This study presents a comprehensive characterization of the AMT gene family in flowering Chinese cabbage and highlights that BcAMT1.1 is a high-affinity ammonium transporter. *BcAMT1.1* is strongly induced by nitrogen starvation or low-NH_4_^+^ levels and is repressed at higher NH_4_^+^ levels, indicating its key role in adjusting nitrogen acquisition to external nitrogen availability. Heterologous overexpression of *BcAMT1.1* in *Arabidopsis* enhanced plant growth under low-nitrogen conditions and increased NH_4_^+^ influx. When NO_3_^−^ and NH_4_^+^ coexist, overexpressing *BcAMT1.1* influences the uptake of two nitrogen forms, altering the expression of nitrogen assimilation-related genes. Based on these findings, we propose a mechanistic model in which BcAMT1.1 regulates nitrogen uptake and assimilation under low-nitrogen conditions, with CIPK23 potentially contributing to the coordination of NH_4_^+^/NO_3_^−^ uptake and assimilation (Figure 12). Despite this, functional analyses of BcAMT1.1 were performed primarily in *Arabidopsis* seedlings, and its specific roles in flowering Chinese cabbage, particularly in nitrate accumulation and nitrogen distribution within edible organs, require further clarification. Future studies will generate both overexpression and CRISPR-cas9 knockout lines in flowering Chinese cabbage to validate the function of BcAMT1.1 under diverse nitrogen regimes and assess its potential effects, guiding strategies for improving nitrogen use efficiency.

## Figures and Tables

**Table 1:** Characteristics of *BcAMT* gene family members in flowering Chinese cabbage.

Gene ID	Gene Name	Chr	Start	End	MW (kDa)	pI	AA (aa)	Instability Index	GRAVY	TM	Subcellular Localization	Category
Bra_cxA05g029310.1	*BcAMT1.1*	A05	9051433	9052957	53.62	7.13	503	25.33	0.38	9	Cell membrane	AMT1
Bra_cxA09g068650.1	*BcAMT1.2*	A09	1262097	1263778	54.88	7.73	512	24.10	0.35	10	Cell membrane	AMT1
Bra_cxA07g035530.1	*BcAMT1.3*	A07	8638823	8640466	54.11	6.74	504	28.46	0.35	9	Cell membrane	AMT1
Bra_cxA01g016480.1	*BcAMT1.3-like*	A01	20920458	20926484	50.79	5.87	476	26.46	0.39	10	Cell membrane	AMT1
Bra_cxA01g038520.1	*BcAMT1.4*	A01	4314030	4315548	53.66	5.7	501	26.06	0.43	10	Cell membrane	AMT1
Bra_cxA03g011620.1	*BcAMT1.4-like*	A03	28109231	28110768	54.41	5.45	509	27.01	0.45	10	Cell membrane	AMT1
Bra_cxA03g025790.1	*BcAMT1.5*	A03	20119522	20121038	53.17	5.96	500	25.32	0.43	10	Cell membrane	AMT1
Bra_cxA05g037880.1	*BcAMT2.1*	A05	3982245	3986822	52.63	6.32	489	28.53	0.45	11	Cell membrane	AMT2
Bra_cxA04g005660.1	*BcAMT2.1-like*	A04	23713465	23716475	52.52	7.28	488	25.86	0.45	11	Cell membrane	AMT2

AA: amino acids; Chr: chromosome; GRAVY: grand average of hydropathy; MW: molecular weight; pI: isoelectric point; TM: transmembrane.

**Table 2:** Divergence time estimation for paralogous gene pairs of *BcAMTs*.

Seq_1	Seq_2	Identity (%)	Ka	Ks	Ka/Ks	T/(MYA)
BcAMT1.3	BcAMT1.3-like	87.30	0.0394	0.4100	0.0962	13.6678
BcAMT1.5	BcAMT1.3	84.33	0.0903	0.6636	0.1360	22.1207
BcAMT1.5	BcAMT1.3-like	81.04	0.0782	0.7431	0.1053	24.7684
BcAMT1.4	BcAMT1.4-like	90.57	0.0454	0.4027	0.1129	13.4226
BcAMT2.1	BcAMT2.1-like	92.84	0.0438	0.3165	0.1383	10.5503

Ks: synonymous substitution rate; Ka: nonsynonymous substitution rate; T: divergence time.

## Data Availability

The original contributions presented in this study are included in the article/Supplementary Material. Further inquiries can be directed to the corresponding author.

## Supplementary Materials

The following supporting information can be downloaded at https://www.mdpi.com/article/10.3390/plants14243812/s1. Table S1. The sequence information for each AMT protein for phylogenetic analysis; Table S2. All gene pairs of three different genomes (*Brassica campestris vs. Arabidopsis thaliana*, and *Brassica campestris vs. Brassica napus*); Table S3: Protein–protein interaction network prediction of BcAMT1.1; Table S4: The primers used in this study. Figure S1: Identification of qRT-PCR in different *BcAMT1.1*-overexpressing lines; Figure S2: Growth phenotype and fresh weight of overexpressing *BcAMT1.1* on NH_4_^+^ toxic analog methylammonium (MeA); Figure S3: Expression of nitrate transporter genes in roots and shoots of wildtype and overexpressing *BcAMT1.1* lines.
