# Supplementary material for: Genome-Wide Analysis of Ammonium Transporter Genes in Flowering Chinese Cabbage and Functional Insights into BcAMT1.1 Under Low-Nitrogen Conditions
Source: Plants (Basel). 2025 Dec 14;14(24):3812. doi: 10.3390/plants14243812 (PMC12737060; doi:10.3390/plants14243812)
Supplement: Supplementary file 1 [file plants-14-03812-s001.zip › Revised Supplementary materials 12.3.pdf]

Genome-Wide Analysis of Ammonium Transporter Genes in Flowering Chinese Cabbage and Functional Insights into BcAMT1.1 under Low-Nitrogen Conditions

Table S1. The sequence information for each AMT protein used for the phylogenetic analysis.

Species	Protein name	Accession numbers
Flowering Chinese cabbage	BcAMT1.1	Bra_cxA05g029310.1
(*Brassica campestris* L. ssp.	BcAMT1.2	Bra_cxA09g068650.1
chinensis var. *utilis* Tsen et Lee)	BcAMT1.3	Bra_cxA07g035530.1
in NGDC	BcAMT1.3-like	Bra_cxA01g016480.1
	BcAMT1.4	Bra_cxA01g038520.1
	BcAMT1.4-like	Bra_cxA03g011620.1
	BcAMT1.5	Bra_cxA03g025790.1
	BcAMT2.1	Bra_cxA05g037880.1
	BcAMT2.1-like	Bra_cxA04g005660.1
Arabidopsis thaliana in BRAD	AtAMT1.1	AT4G13510.1
	AtAMT1.2	AT1G64780.1
	AtAMT1.3	AT3G24300.1
	AtAMT1.4	AT4G28700.1
	AtAMT1.5	AT3G24290.1
	AtAMT2.1	AT2G38290.1
Brassica napus in Genoscope	BnAMT1.1a	BnaC08g08610D
	BnAMT1.1b	BnaC06g11810D
	BnAMT1.1c	BnaA05g35560D
	BnAMT1.2a	BnaCnng01740D
	BnAMT1.2b	BnaA09g00320D
	BnAMT1.3a	BnaA07g05760D
	BnAMT1.3b	BnaUnng02430D
	BnAMT1.3c	BnaA01g23190D
	BnAMT1.4a	BnaC07g41470D
	BnAMT1.4b	BnaC01g09770D
	BnAMT1.4c	BnaA01g08220D
	BnAMT1.5a	BnaA03g37270D
	BnAMT1.5b	BnaC03g42390D
	BnAMT1.5c	BnaC03g74280D
	BnAMT2.1a	BnaA05g06450D
	BnAMT2.1b	BnaC04g07100D
	BnAMT2.1c	BnaC04g07090D
	BnAMT2.2a	BnaCnng62050D
	BnAMT2.2b	BnaC04g56650D
	BnAMT2.2c	BnaA04g21900D

Continued Table S1.

Solanum lycopersicum in GenBank	SlAMT1.1	NP_001304667.1
	SlAMT1.2	NP_001234253.2
	SlAMT1.3	NP_001234216.1
Nicotiana tabacum in GenBank	NtAMT1.1	XP_009784484.1
	NtAMT1.2	XP_009795683.1
	NtAMT1.3	XP_009768027.1
	NtAMT2.1	XP_009778221.1
	NtAMT3.1	XP_009800812.1
	NtAMT4.1	XP_009799644.1
	NtAMT4.2	XP_009784746.1
	NtAMT4.3	XP_009796398.1
	NtAMT4.4	XP_009765535.1
Oryza sativa in GenBank	OsAMT1.1	CAE03364.1
	OsAMT1.2	BAD21532.1
	OsAMT1.3	BAD21574.1
	OsAMT2.1	BAC65231.1
	OsAMT2.2	CAY33634.1
	OsAMT2.3	NP_915334.1
	OsAMT3.1	BAD33268.1
	OsAMT3.2	BAD33268.1
	OsAMT3.3	AAO41130.1
	OsAMT4.1	Q10CV4.1
Populus trichocarpa in UniProt	PtrAMT1.1	B9HSW3
	PtrAMT1.2	B9IPE2
	PtrAMT1.3	B9HKW8
	PtrAMT1.4	B9GRB5
	PtrAMT1.5	B9GRB4
	PtrAMT1.6	B9HP47
	PtrAMT2.1	B9HCZ0
	PtrAMT2.2	B9IGE2
	PtrAMT3.1	B9GHA5
	PtrAMT4.1	B9GS88
	PtrAMT4.2	B9IKS2
	PtrAMT4.3	B9H8E7
	PtrAMT4.4	B9I5F0

Table S2. All gene pairs of three different genomes (*Brassica campestris* vs *Arabidopsis thaliana*, and *Brassica campestris* vs *Brassica napus*).

BcAMT-AtAMT		BcAMT-BnAMT			
BcAMT1.3	AtAMT1.5	BcAMT1.1	BnaAMT1.1b	BcAMT1.1	BnaAMT1.1b
BcAMT1.3	AtAMT1.1	BcAMT1.2	BnaAMT1.2a	BcAMT1.2	BnaAMT1.2a
BcAMT1.3-like	AtAMT1.5	BcAMT1.2	BnaAMT1.2b	BcAMT1.2	BnaAMT1.2b
BcAMT1.3-like	AtAMT1.1	BcAMT1.3	BnaAMT1.3a	BcAMT1.3	BnaAMT1.3a
BcAMT1.4	AtAMT1.4	BcAMT1.3	BnaAMT1.5a	BcAMT1.3-like	BnaAMT1.3a
BcAMT1.4-like	AtAMT1.4	BcAMT1.3-like	BnaAMT1.3a	BcAMT1.5	BnaAMT1.3a
BcAMT1.5	AtAMT1.5	BcAMT1.3-like	BnaAMT1.5a	BcAMT1.4	BnaAMT1.4a
BcAMT1.5	AtAMT1.1	BcAMT1.4	BnaAMT1.4a	BcAMT1.4-like	BnaAMT1.4a
BcAMT2.1	AtAMT2.1	BcAMT1.4	BnaAMT1.4b	BcAMT1.4	BnaAMT1.4b
BcAMT2.1-like	AtAMT2.1	BcAMT1.4	BnaAMT1.4c	BcAMT1.4-like	BnaAMT1.4b
		BcAMT1.4-like	BnaAMT1.4a	BcAMT1.4	BnaAMT1.4c
		BcAMT1.4-like	BnaAMT1.4b	BcAMT1.4-like	BnaAMT1.4c
		BcAMT1.4-like	BnaAMT1.4c	BcAMT1.3	BnaAMT1.5a
		BcAMT1.5	BnaAMT1.3a	BcAMT1.3-like	BnaAMT1.5a
		BcAMT1.5	BnaAMT1.5a	BcAMT1.5	BnaAMT1.5a
		BcAMT2.1	BnaAMT2.1a	BcAMT2.1	BnaAMT2.1a
		BcAMT2.1	BnaAMT2.1c	BcAMT2.1-like	BnaAMT2.1a
		BcAMT2.1	BnaAMT2.2c	BcAMT2.1	BnaAMT2.1c
		BcAMT2.1-like	BnaAMT2.1a	BcAMT2.1-like	BnaAMT2.1c
		BcAMT2.1-like	BnaAMT2.1c	BcAMT2.1	BnaAMT2.2c
		BcAMT2.1-like	BnaAMT2.2c	BcAMT2.1-like	BnaAMT2.2c

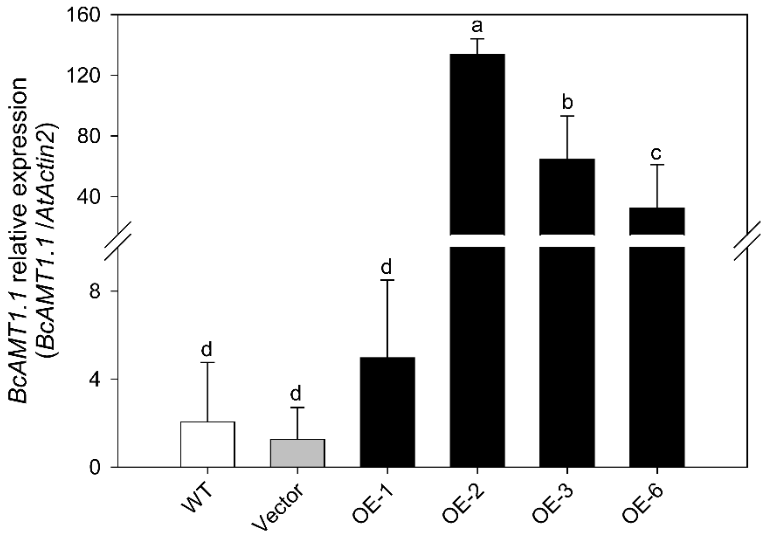

Figure S1. Identification of qRT-PCR in different *BcAMT1.1*-overexpressing lines. WT: wild-type; OE-1, OE-2, OE-3, and OE-6 represent overexpressing *BcAMT1.1* lines 1, 2, 3, and 6, respectively. Bars indicate mean ± SD ($n = 3$), different letters above the bars indicate significant differences at $p < 0.05$.

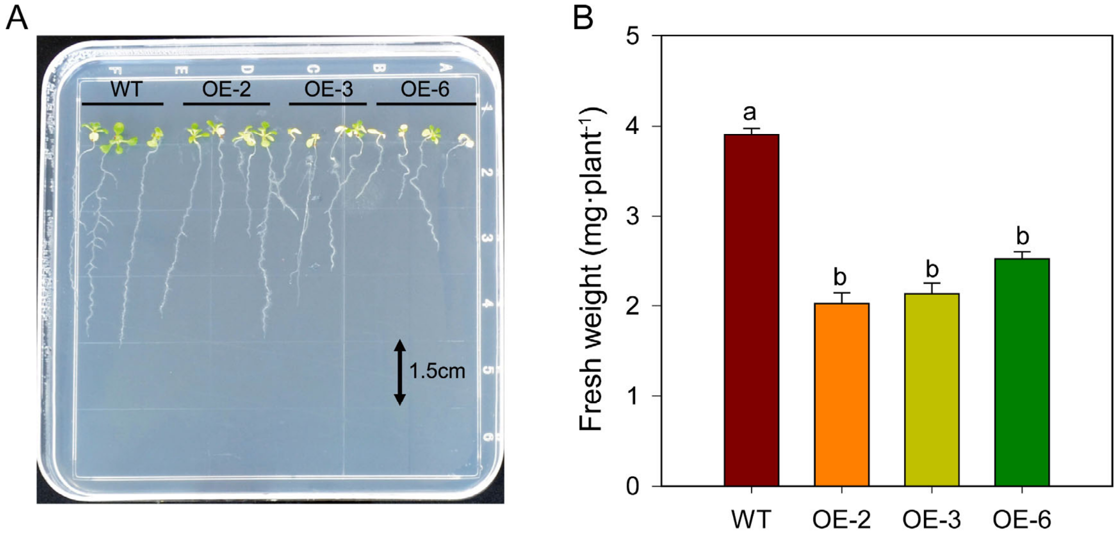

Figure S2. Growth phenotype and fresh weight of overexpressing *BcAMT1.1* on NH_4^+ toxic analog methylammonium (MeA). (**A**) Growth of wildtype (WT) and overexpression lines (line 2, 3, and 6) on agarose containing 20 mmol·L^{-1} MeA for 10 d aftera 4 d pre-culture on 4 mmol·L^{-1} NO_3^-. (**B**) Fresh weight of whole plants of WT and overexpression lines. WT: wildtype; OE-2, OE-3, and OE-6 represent overexpressing *BcAMT1.1* lines 2, 3, and 6, respectively. Data represent mean ± SD (*n*=10), different letters above bars indicate significant differences at $p < 0.05$.

Table S3. Protein–protein interaction network prediction of BcAMT1.1.

Node1	Node2	Node1 String ID	Node2 String ID	Neighborhood on chromosome	Gene fusion	Phylogenetic cooccurrence	Homology	Coexpression	Experimentally determined interaction	Database annotated	Automated textmining	Combined score
BcAMT1.1	NRT2.1	3702.P54144	3702.O82811	0	0	0	0	0.08	0	0	0.8	0.808
BcAMT1.1	GLN1.4	3702.P54144	3702.Q9FMD9	0.098	0	0	0	0.287	0	0	0.686	0.78
BcAMT1.1	GLN2	3702.P54144	3702.Q43127	0.098	0	0	0	0.257	0	0	0.704	0.784
BcAMT1.1	NRT2.4	3702.P54144	3702.Q9FJH8	0	0	0	0	0.08	0	0	0.784	0.793
BcAMT1.1	GLN1.3	3702.P54144	3702.Q9LVI8	0.098	0	0	0	0.257	0	0	0.738	0.809
BcAMT1.1	NPF6.3	3702.P54144	3702.Q05085	0	0	0	0	0.054	0	0	0.828	0.83
BcAMT1.1	GLN1.1	3702.P54144	3702.Q56WN1	0.098	0	0	0	0.344	0	0	0.754	0.842
BcAMT1.1	AMT1.3	3702.P54144	3702.Q9SQH9	0	0	0.05	0.981	0	0.483	0.72	0.059	0.853
BcAMT1.1	CIPK23	3702.P54144	3702.Q93VD3	0	0	0	0	0	0	0	0.874	0.874
BcAMT1.1	GLB1	3702.P54144	3702.Q9ZST4	0.122	0	0.308	0	0.751	0.821	0	0.321	0.978
AMT1.3	NRT2.1	3702.Q9SQH9	3702.O82811	0	0	0	0	0.1	0	0	0.628	0.651
AMT1.3	NPF6.3	3702.Q9SQH9	3702.Q05085	0	0	0	0	0	0	0	0.812	0.812
AMT1.3	GLN2	3702.Q9SQH9	3702.Q43127	0.098	0	0	0	0.257	0	0	0.682	0.768
AMT1.3	GLN1.1	3702.Q9SQH9	3702.Q56WN1	0.098	0	0	0	0.257	0	0	0.626	0.728
AMT1.3	CIPK23	3702.Q9SQH9	3702.Q93VD3	0	0	0	0	0.069	0	0	0.776	0.782
AMT1.3	NRT2.4	3702.Q9SQH9	3702.Q9FJH8	0	0	0	0	0.106	0	0	0.542	0.573
AMT1.3	GLN1.4	3702.Q9SQH9	3702.Q9FMD9	0.098	0	0	0	0.257	0	0	0.697	0.779
AMT1.3	GLN1.3	3702.Q9SQH9	3702.Q9LVI8	0.098	0	0	0	0.278	0	0	0.65	0.752
AMT1.3	GLB1	3702.Q9SQH9	3702.Q9ZST4	0.122	0	0.302	0	0.751	0.821	0	0.321	0.978
CIPK23	NRT2.1	3702.Q93VD3	3702.O82811	0	0	0	0	0	0	0	0.868	0.868
CIPK23	NPF6.3	3702.Q93VD3	3702.Q05085	0	0	0	0	0	0.237	0	0.926	0.941
CIPK23	NRT2.4	3702.Q93VD3	3702.Q9FJH8	0	0	0	0	0	0	0	0.667	0.667

Continued Table S3.

GLB1	GLN2	3702.Q9ZST4	3702.Q43127	0.12	0	0	0	0.27	0	0.122	0.37	0.597
GLB1	GLN1.1	3702.Q9ZST4	3702.Q56WN1	0.12	0	0	0	0.27	0	0.122	0.373	0.599
GLB1	GLN1.4	3702.Q9ZST4	3702.Q9FMD9	0.12	0	0	0	0.27	0	0.122	0.309	0.558
GLB1	GLN1.3	3702.Q9ZST4	3702.Q9LVI8	0.12	0	0	0	0.27	0	0.122	0.309	0.558
GLN1.1	NRT2.1	3702.Q56WN1	3702.O82811	0	0	0	0	0.054	0	0	0.809	0.812
GLN1.1	NPF6.3	3702.Q56WN1	3702.Q05085	0	0	0	0	0.093	0	0	0.886	0.892
GLN1.1	GLN2	3702.Q56WN1	3702.Q43127	0	0	0.05	0.98	0	0	0.6	0.046	0.606
GLN1.1	GLN1.3	3702.Q56WN1	3702.Q9LVI8	0	0	0.048	0.984	0.06	0	0.6	0.047	0.613
GLN1.1	GLN1.4	3702.Q56WN1	3702.Q9FMD9	0	0	0.048	0.985	0.129	0	0.6	0.045	0.641
GLN1.1	NRT2.4	3702.Q56WN1	3702.Q9FJH8	0	0	0	0	0	0	0	0.751	0.751
GLN1.3	NRT2.1	3702.Q9LVI8	3702.O82811	0	0	0	0	0	0	0	0.802	0.802
GLN1.3	NPF6.3	3702.Q9LVI8	3702.Q05085	0	0	0	0	0.073	0	0	0.882	0.885
GLN1.3	GLN2	3702.Q9LVI8	3702.Q43127	0	0	0.051	0.978	0	0	0.6	0.042	0.604
GLN1.3	NRT2.4	3702.Q9LVI8	3702.Q9FJH8	0	0	0	0	0	0	0	0.75	0.75
GLN1.3	GLN1.4	3702.Q9LVI8	3702.Q9FMD9	0	0	0.049	0.982	0	0	0.6	0.047	0.606
GLN1.4	NRT2.1	3702.Q9FMD9	3702.O82811	0	0	0	0	0.047	0	0	0.808	0.809
GLN1.4	NPF6.3	3702.Q9FMD9	3702.Q05085	0	0	0	0	0.073	0	0	0.89	0.893
GLN1.4	GLN2	3702.Q9FMD9	3702.Q43127	0	0	0.05	0.98	0	0	0.6	0.043	0.604
GLN1.4	NRT2.4	3702.Q9FMD9	3702.Q9FJH8	0	0	0	0	0.045	0	0	0.75	0.751
GLN2	NRT2.1	3702.Q43127	3702.O82811	0	0	0	0	0	0	0	0.809	0.809
GLN2	NPF6.3	3702.Q43127	3702.Q05085	0	0	0	0	0.081	0	0	0.89	0.894
GLN2	NRT2.4	3702.Q43127	3702.Q9FJH8	0	0	0	0	0	0	0	0.75	0.75
NPF6.3	NRT2.1	3702.Q05085	3702.O82811	0	0	0	0	0.061	0	0	0.961	0.962
NPF6.3	NRT2.4	3702.Q05085	3702.Q9FJH8	0	0	0	0	0.049	0	0	0.908	0.909

Table S4. The primers used in the study.

Primer name	Sequence (5′–3′)	Usage
BcAMT1.1	F: GCAGGTCGACTCTAGAATGTCGGGATCTTTATCTTGC<br>R: ACGAGCTCGGTACCCGGGTCAAACAGAAGTGGTAGTAAC	Cloning *BcAMT1.1* into pCAMBIA3301 vector using *Xba* I and *Sma* I sites
pYES-AMT1.1	F: CAGTGTGCTGGAATTCATGTCGGGATCTTTATCTTGC<br>R: ATGCGGCCCTCTAGATCAAACAGAAGTGGTAGTAAC	Cloning *BcAMT1.1* into pYES2 vector using *EcoR* I and *Xba* I sites
pBI-AMT1.1	F: CACGGGGGACTCTAGAATGTCGGGATCTTTATCTTGC<br>R: TCCTTTACCCATCCCGGGAACAGAAGTGGTAGTAAC	Cloning *BcAMT1.1* into pBI121 vector using *Xba* I and *Sma* I sites
q-BcAMT1.1	F: CTTCAACAAGATCCTCGTCAC<br>R: GTTATAGCTGCAAACCCTCC	qRT-PCR for the cDNA of flowering Chinese cabbage
GAPDH	F: CAGGTTTGGAATTGTCGAGG<br>R: GAGCTGTGGAAGCACCTTTC	
q-AtGLN1.1	F: CAACCTTAACCTCTCAGACTCCACT<br>R: CAGCTGCAACATCAGGGTTGCTA	qRT-PCR for the cDNA of *Arabidopsis*
q-AtGLN1.2	F: TAACCTTGACATCTCAGACAACAGT<br>R: TCAGCAATAACATCAGGGTTAGCA	
q-AtGLN2	F: CCAACATGTCAGATGAGAGTGCC<br>R: CCAGGTGCTTGACCGGTACTCG	
q-AtGDH2	F: AGCTGATCCACGAGAAAGGC<br>R: TGAGACTTCCAGTTGCGTCC	
q-AtGLT1	F: GGTCTTCCATGGGAACTGGG<br>R: TAGCAATTCCCACAGGGCAG	
q-AtNRT1.1	F: CGTCTTTACAATTTCTACTG<br>R: TCATCATCCAACTCAATC	
q-AtNRT2.1	F: GCTTGCACGTTACCTGTGA<br>R: TCCTTGATGCATGTTCTTCTG	
q-ACTIN2	F: TCGGTGGTTCCATTCTTGCT<br>R: GCTTTTTAAGCCTTTGATCTTGAGAG	

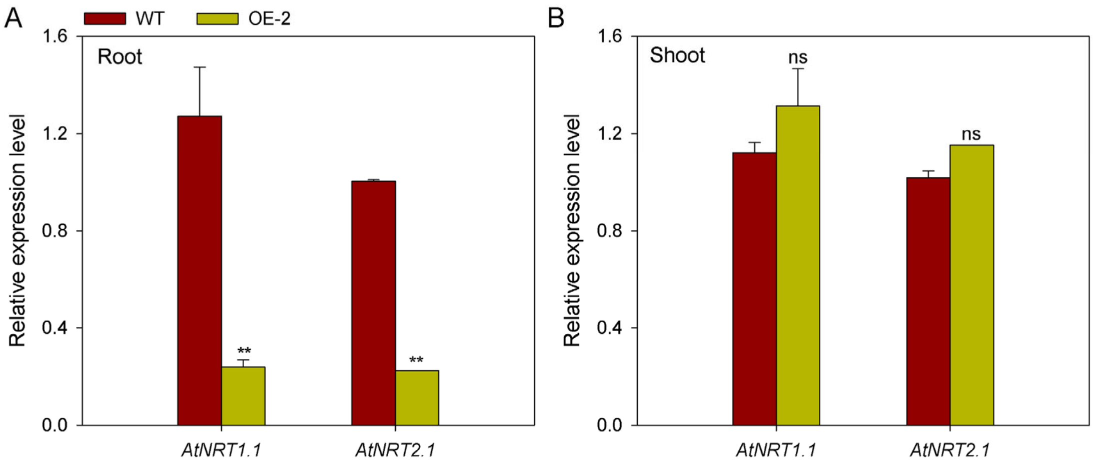

Figure S3: Expression of nitrate transporter genes in roots and shoots of wildtype and overexpressing *BcAMT1.1* lines. The data represent the mean ± SD (n = 3). WT: wildtype; OE-2: overexpression line 2. ns: no significant difference, ** represents significant differences at $P < 0.01$.
