# Supplementary figures and images for: Genome-Wide Analysis of Ammonium Transporter Genes in Flowering Chinese Cabbage and Functional Insights into BcAMT1.1 Under Low-Nitrogen Conditions
Source: Plants (Basel). 2025 Dec 14;14(24):3812. doi: 10.3390/plants14243812 (PMC12737060; doi:10.3390/plants14243812)

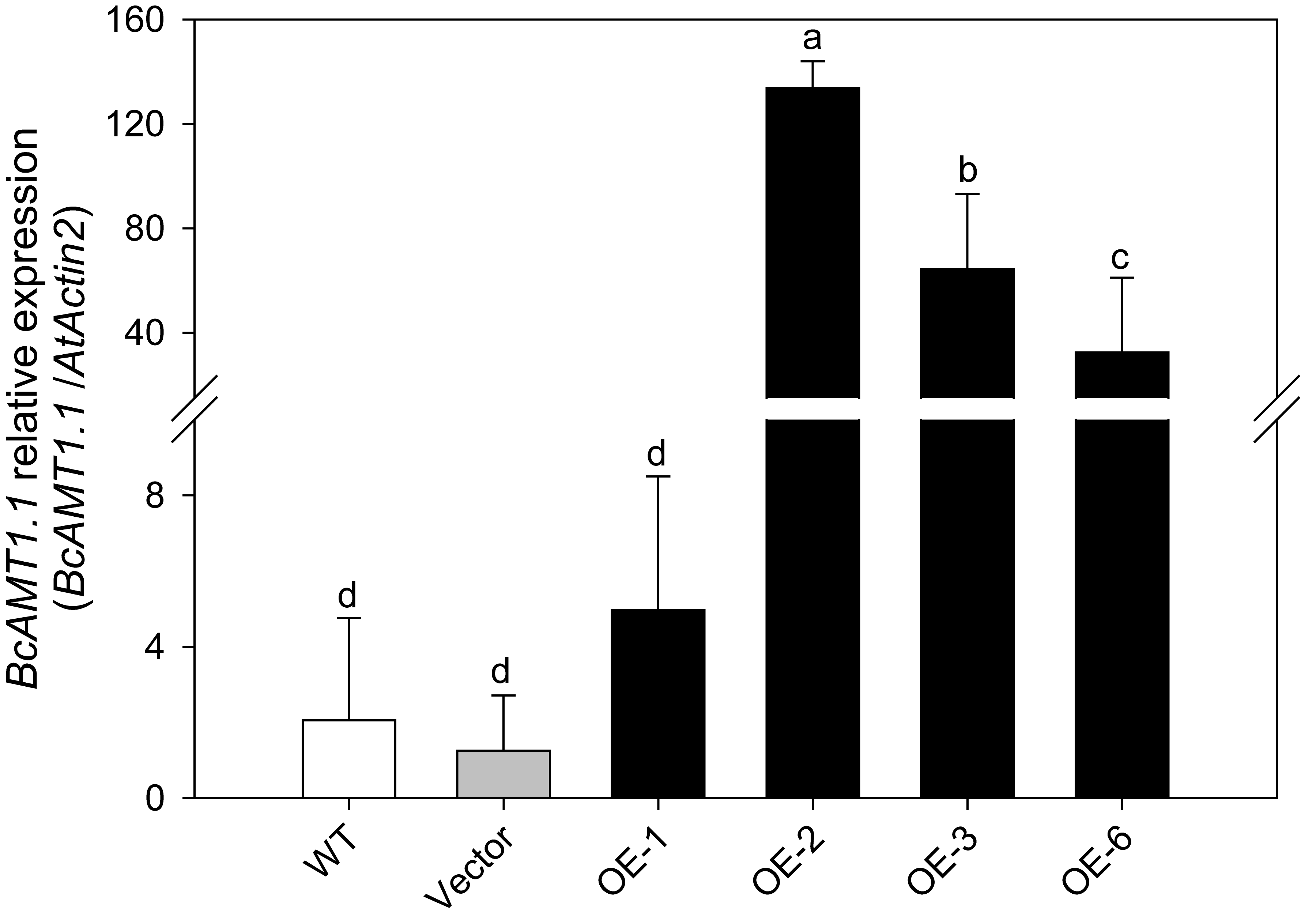

Supplement: Supplementary file 1 [file plants-14-03812-s001.zip › Figure S1.tif]

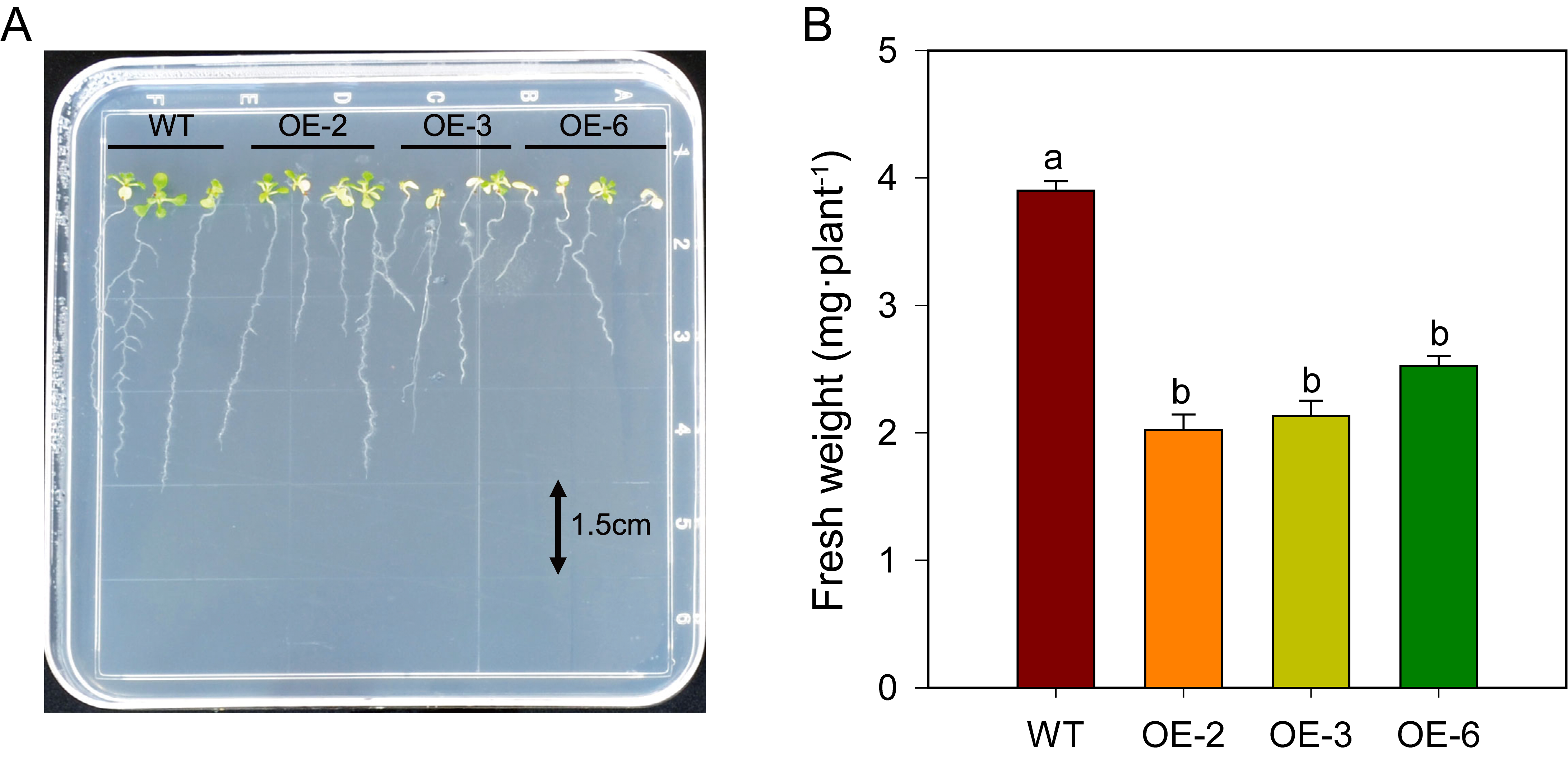

Supplement: Supplementary file 1 [file plants-14-03812-s001.zip › Figure S2.tif]

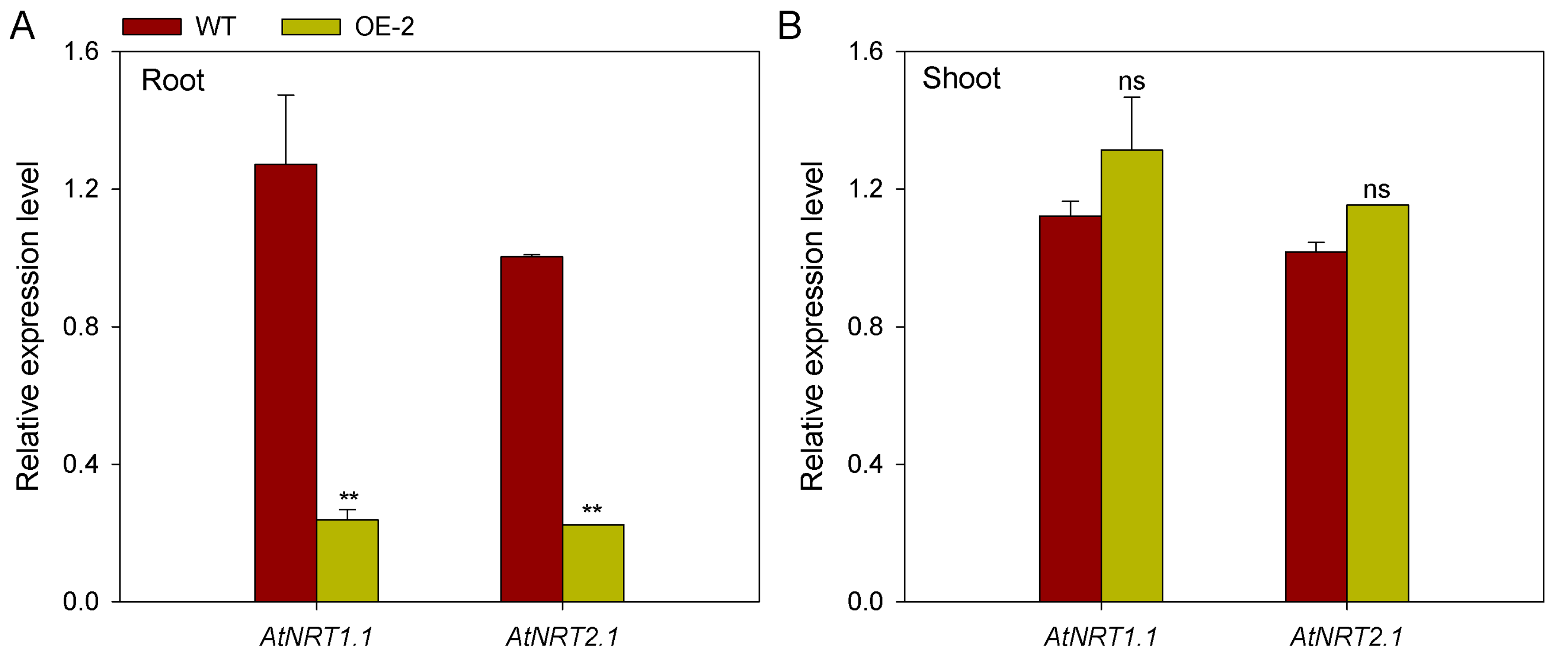

Supplement: Supplementary file 1 [file plants-14-03812-s001.zip › Figure S3.tif]
